# Oral-gut microbial transmission promotes diabetic coronary heart disease

**DOI:** 10.1186/s12933-024-02217-y

**Published:** 2024-04-05

**Authors:** Yiwen Li, Yanfei Liu, Jing Cui, Mengmeng Zhu, Wenting Wang, Keji Chen, Luqi Huang, Yue Liu

**Affiliations:** 1 National Clinical Research Center for TCM Cardiology, Xiyuan Hospital of China Academy of Chinese Medical Sciences, Beijing, 100091 China; 2 Beijing Key Laboratory of Traditional Chinese Medicine Basic Research on Prevention and Treatment for Major Diseases, Experimental Research Center, China Academy of Chinese Medical Sciences, Beijing, 100078 China; 3 China Academy of Chinese Medical Sciences, Beijing, 100078 China

**Keywords:** Diabetic coronary heart disease, Oral microbiota, Oral-gut axis, *Fusobacterium nucleatum*, Myocardial ischemia–reperfusion injury

## Abstract

**Background:**

Diabetes is a predominant driver of coronary artery disease worldwide. This study aims to unravel the distinct characteristics of oral and gut microbiota in diabetic coronary heart disease (DCHD). Simultaneously, we aim to establish a causal link between the diabetes-driven oral-gut microbiota axis and increased susceptibility to diabetic myocardial ischemia–reperfusion injury (MIRI).

**Methods:**

We comprehensively investigated the microbial landscape in the oral and gut microbiota in DCHD using a discovery cohort (n = 183) and a validation chohort (n = 68). Systematically obtained oral (tongue-coating) and fecal specimens were subjected to metagenomic sequencing and qPCR analysis, respectively, to holistically characterize the microbial consortia. Next, we induced diabetic MIRI by administering streptozotocin to C57BL/6 mice and subsequently investigated the potential mechanisms of the oral-gut microbiota axis through antibiotic pre-treatment followed by gavage with specific bacterial strains (*Fusobacterium nucleatum* or fecal microbiota from DCHD patients) to C57BL/6 mice.

**Results:**

Specific microbial signatures such as oral *Fusobacterium nucleatum* and gut *Lactobacillus, Eubacterium,* and *Roseburia faecis*, were identified as potential microbial biomarkers in DCHD. We further validated that oral *Fusobacterium nucleatum* and gut *Lactobacillus* are increased in DCHD patients, with a positive correlation between the two. Experimental evidence revealed that in hyperglycemic mice, augmented *Fusobacterium nucleatum* levels in the oral cavity were accompanied by an imbalance in the oral-gut axis, characterized by an increased coexistence of *Fusobacterium nucleatum* and *Lactobacillus*, along with elevated cardiac miRNA-21 and a greater extent of myocardial damage indicated by TTC, HE, TUNEL staining, all of which contributed to exacerbated MIRI.

**Conclusion:**

Our findings not only uncover dysregulation of the oral-gut microbiota axis in diabetes patients but also highlight the pivotal intermediary role of the increased abundance of oral *F. nucleatum* and gut *Lactobacillus* in exacerbating MIRI. Targeting the oral-gut microbiota axis emerges as a potent strategy for preventing and treating DCHD. Oral-gut microbial transmission constitutes an intermediate mechanism by which diabetes influences myocardial injury, offering new insights into preventing acute events in diabetic patients with coronary heart disease.

**Graphic Abstract:**

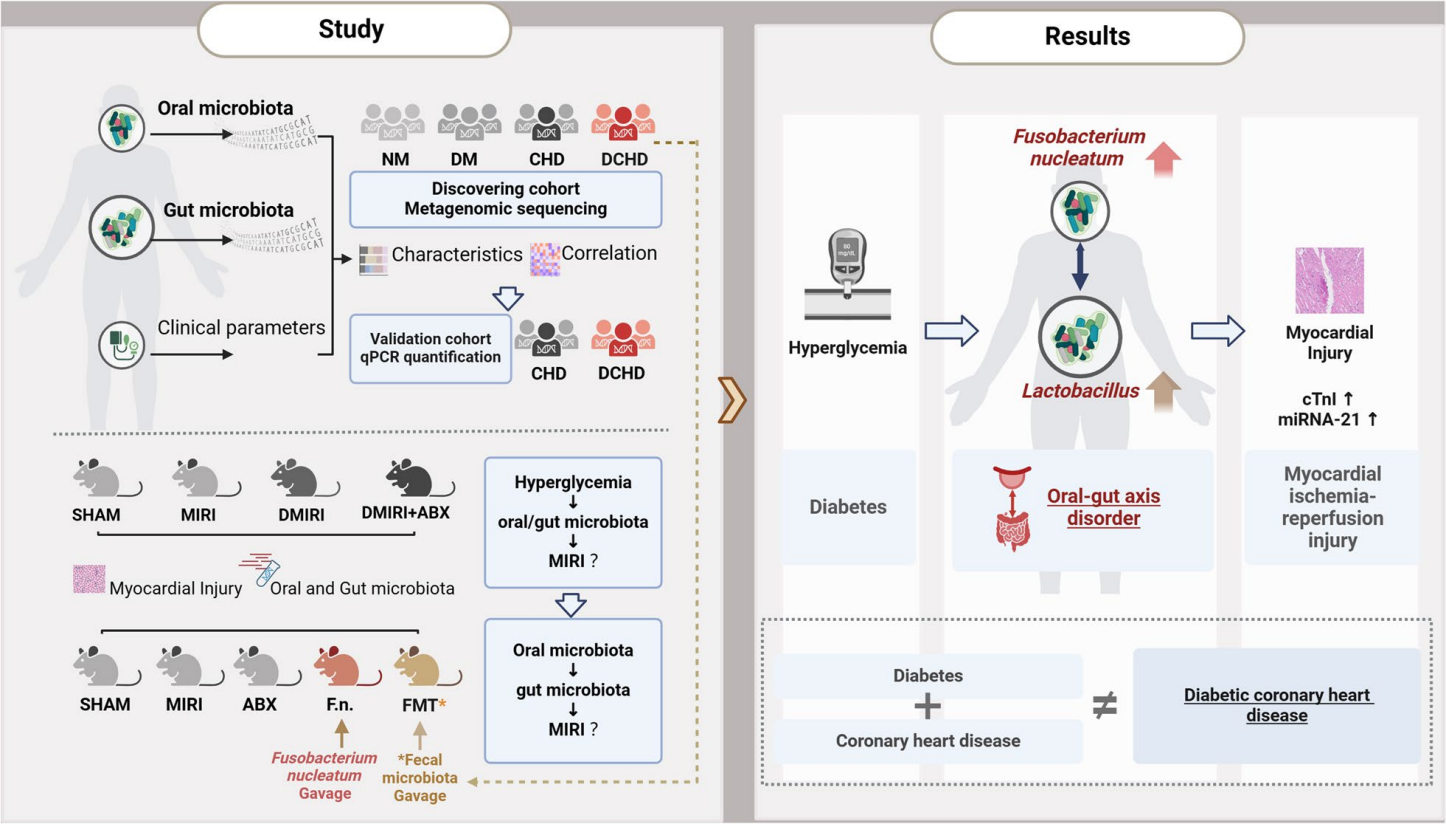

**Supplementary Information:**

The online version contains supplementary material available at 10.1186/s12933-024-02217-y.

## Highlights

- In this study, we found and verified for the first time that the increase in the abundance of oral *Fusobacterium nucleatum* and gut *Lactobacillus* is a unique oral-gut microbiota characteristic of patients with diabetic coronary heart disease (DCHD).
- This study reveals a broad and close association between the oral-gut microbiota in DCHD patients, which may be associated with co-diabetes.
- This study reveals a novel mechanism by which diabetes exacerbates myocardial ischemia–reperfusion injury in coronary heart disease: diabetes increases *F. nucleatum* abundance, resulting in oral-gut microbiota disruption characterized by increased *F. nucleatum-Lactobacillus* abundance, exacerbating myocardial ischemia–reperfusion injury.
- The present investigation is the inaugural to propose that the manipulation of the oral-gut microbiome axis constitutes a potentially pivotal approach in the prevention and management of coronary heart disease in diabetic patients.

**Supplementary Information:**

The online version contains supplementary material available at 10.1186/s12933-024-02217-y.

## Introduction

Metabolic disorders are risk factors for coronary heart disease (CHD) [1]. Individuals diagnosed with diabetes exhibit a heightened incidence and intensified severity of acute coronary syndromes [2, 3]. An increasing number of studies have paid attention to the unique mechanism of cardiometabolic diseases, which result from complex gene-environment interactions [4, 5]. Although there are significant genetic influences on both DM and CHD [6, 7], the macrovascular complications based on those of diabetic coronary heart disease (DCHD) exhibit a high degree of heterogeneity among patients and are strongly associated with microbiome [8, 9]. The gut and oral are the two colonized sites with the most extensive microbial functions in the human body [10–12]. Dysbiosis of these microbiota is linked to insulin resistance and myocardial infarction [13, 14]. And is intimately involved in the disease process of myocardial infarction [15, 16].

Although the number of shared taxa between oral and gut microbiota is limited due to the gastric bactericidal barrier [17], intestinal motility, or bile and pancreatic secretions, there is a close association between oral and gut microbiota. Oral-gut microbial transmission contributes to elucidating the aggravation of CHD caused by DM [18, 19]. Some studies have searched for several potential oral and gut pathogenic microbiota, which may lead to periodontitis [20] and leaky gut [21] and are strongly associated with systemic inflammatory diseases [9, 22]. Studies have focused on the relationship between specific species of microbiota and the host, but there is a lack of focus on the community relationship within the microbiota. Focusing on oral-gut microbiota transmission helps further understand the mechanisms by which DM promotes the development of cardiovascular disease and provides a theoretical basis for risk assessment and prevention strategies of DCHD.

In this study, we investigated the role of the oral/gut microbiota and oral-gut microbial transmission in DCHD. Our study specifically targeted the tongue coating microbiota (oral microbiota), given its critical role in tongue diagnosis in traditional Chinese medicine (TCM). Through two cohort studies, we screened and validated the characterization of oral and gut microbiota in DCHD by metagenomic sequencing, and gained preliminary insights into the relationships between oral and gut microbiota and the correlations between oral-gut microbiota and cardiovascular metabolism-related markers. We initially identified a synchronized increase of *Fusobacterium nucleatum* in oral perfringens and *Lactobacillus* in the gut as the oral-gut microbial signature of DCHD. To verify whether diabetes promotes this oral-gut microbiota disturbance and further aggravates myocardial injury, we confirmed the causal relationship between diabetes promotion of oral-gut microbial disturbance as well as disturbed oral-gut microbial transmission and DCHD through animal experiments. Therefore, we confirmed that DM promotes oral-gut microbiota disturbance, which further aggravates CHD. The mechanism of *F. nucleatum* on cardiovascular disease merits further investigation. It further enhances the understanding of the scientific basis underlying tongue diagnosis in TCM.

## Methods

### Cohort information

The study participants of the discovery cohort were patients who were outpatients or inpatients in Xiyuan Hospital of China Academy of Chinese Medical Sciences. This study was approved by the Ethics Committee of Xiyuan Hospital of China Academy of Chinese Medical Sciences (2021XLA046-2). Trial registration: ChiCTR2100050559. All participants provided written informed consent. The participants were divided into four groups: (1) normal (NM), n = 36; (2) DM, n = 33; (3) CHD, n = 57; (4) DCHD, n = 57. To maximize the consistency of severity in patients with CHD, we included patients with CHD history of previous acute coronary syndrome. Detection of oral (tongue coating) and gut (fecal) microbiota was performed using metagenomic sequencing. The raw metagenomic shotgun sequencing data reported in this study are available from the Genome Sequence Archive (GSA) in National Genomics Data Center, Beijing Institute of Genomics (China National Center for Bioinformation), Chinese Academy of Sciences under the accession code CRA015579. Sample collection methods are summarized in the Additional file 2. DCHD is defined as CHD occurring on the basis of metabolic disorders such as DM [23]. The detailed criteria for the diagnosis of DM [24] and CHD [25], inclusion and exclusion criteria, are summarized in the Additional file 2**.**

We had a separate validation cohort. The source of participants, diagnostic criteria, and inclusion and exclusion criteria of the validation cohort were consistent with those of the discovery cohort. The participants were divided into two groups: (1) CHD, n = 33; (2) DCHD, n = 35. The detection of the oral and gut microbiota was performed through qPCR quantification. The screening criteria for the strains were: Differential strains screened in discovery cohort (*F. nucleatum*, *Lactobacillus, Eubacterium,* and *Eubacterium rectale*). The same sequence assay was implemented in tongue coating and fecal samples separately to explore whether there is an oral-gut ectopic colonization of microbiota.

### Microbiome sequencing

Oral and gut samples were collected and DNA was extracted. Metagenomic sequencing was performed at Novogene Bio Inc., Beijing, China and Jiaan weikang Bio Inc., Beijing, China using Illumina platforms. Bioinformatics analysis was performed by our research team (Beijing Institutes of Life Science, Chinese Academy of Sciences). qPCR quantification were performed at Allwegene Bio Inc. Details are summarized in the Additional file 2.

### Metagenomic analysis

The computation of α diversity and β diversity was conducted using the vegdist function from the "vegan" R package [26]. Disparities in the abundance of phylum, genus, and species between any two groups were scrutinized through the Wilcoxon rank-sum test. To address multiple comparisons, a false discovery rate (FDR) correction was applied, employing the Benjamini–Hochberg method. The distinct KEGG were enriched with “clusterProfiler” R package [27]. Spearman correlation analysis were visualized using the “igraph” R package [28]. Oral-gut microbiota tracing analysis used FEAST methods [29].

### qPCR quantification

Total DNA of microbiota was isolated using the Trizol reagent (Tiangen, Beijing, China) following the manufacturer's instruction, PCR amplification using the 2 × Taq MasterMix (CWBio, Beijing, China). After TA cloning, positive clones were identified by colony PCR, TBGreen®Premix ExTaq™II (TliRNaseHPlus), ROXplus (TaKaRa, Japan) in a ABI7500 real-time PCR system (Applied Biosystems, Inc., USA). The primer sequences are summarized in the Additional file 2.

### Animal experiments

A total of two animal experiments were performed. Six-week-old male C57BL/6 J mice were used for all animal experiments and housed in animal facilities with specific SPF levels. All mice were housed under standard conditions (air humidity 40%–70%, ambient temperature 22 ± 2 °C, and 12/12 h light/dark cycle). Mice were purchased from Spectrum (Beijing) Biotechnology Co. Ltd (Production license: SCXK (Beijing) 2019–0010). Experiment I: Establishment of a diabetic model through streptozotocin (STZ) injection, depletion of gut microbiota using a broad-spectrum antibiotic cocktail (ABX) followed by left anterior descending coronary artery ligation to create a myocardial ischemia–reperfusion injury (MIRI) model. Experiment II: After one week of gut microbiota depletion through ABX gavage, the mice underwent single-bacterial gavage (*F. nucleatum*) or human fecal microbiota transplantation (FMT) to reconstruct the intestinal microbiota. Subsequently, left anterior descending coronary artery ligation was performed to establish the myocardial ischemia–reperfusion injury model. Microbiota transplantation protocol: For the first two weeks, gavage was administered every other day, followed by a once-a-week administration for the next four weeks to maintain microbial colonization. For the *F. nucleatum* gavage group: Gavage with 200 μL of *F. nucleatum* bacterial solution. For the FMT group: Gavage with 200 μL of fecal microbiota solution from DCHD patients. Other control groups: Gavage with an equal volume of phosphate-buffered saline. All procedures were conducted using sterilized instruments. A MIRI model was established by inducing myocardial ischemia for 30 min followed by 24 h of reperfusion. This study was approved by the Ethics Committee for Animal Experiments (2022XLC058). Details are summarized in the Additional file 2.

### Microbiota transplantation for mice

Construction of a Pseudo-sterile Mouse Model: Broad-spectrum antibiotics (ampicillin 1 g/L, neomycin sulfate 1 g/L, metronidazole 1 g/L, vancomycin 0.5 g/L) were administered via gavage, 200 µL/day for consecutive 7 days. This antibiotic regimen is designed to eliminate the mouse microbiota within the specified period, and the model is considered successfully prepared after completion [30].

Bacterial Strain Cultivation [31]: *Fusobacterium nucleatum* was cultured in a thioglycolate liquid medium. Prepare a test tube with approximately 10 mL of liquid medium (previously placed in an anaerobic environment for 24 h); disinfect the surface of the ampoule, open it in a safety cabinet, burn the top with an alcohol lamp, quickly add sterile water to rupture it, and then use forceps to open it; draw about 0.5 mL of liquid medium into a freeze-dried tube, dissolve it thoroughly, draw it back into the test tube with liquid medium, and mix well. Place the liquid test tube under specified anaerobic conditions for cultivation. When the bacterial solution becomes turbid and reaches a concentration of 10^9 colony-forming units, it is ready for gavage.

### Statistical analysis

A student’s t-test, a two-way ANOVA followed by Sidak’s multiple comparison test, and a Mann–Whitney U rank-sum test were performed using GraphPad Prism (V9.5) and IBM SPSS Statistics (V26.0). Pearson’s chi-squared test was used for the statistical analysis of sex, drinkers, and follow-up rate between groups. The Adonis test was also performed using the R software. The Kruskal–Wallis rank-sum test was used to analyze the abundance of microbiota. Spearman’s correlations among microbiota, clinical parameters, and metabolites were tested and visualized using the R package.

## Results

### Baseline clinical characteristics of the study cohort

**Fig. 1:**
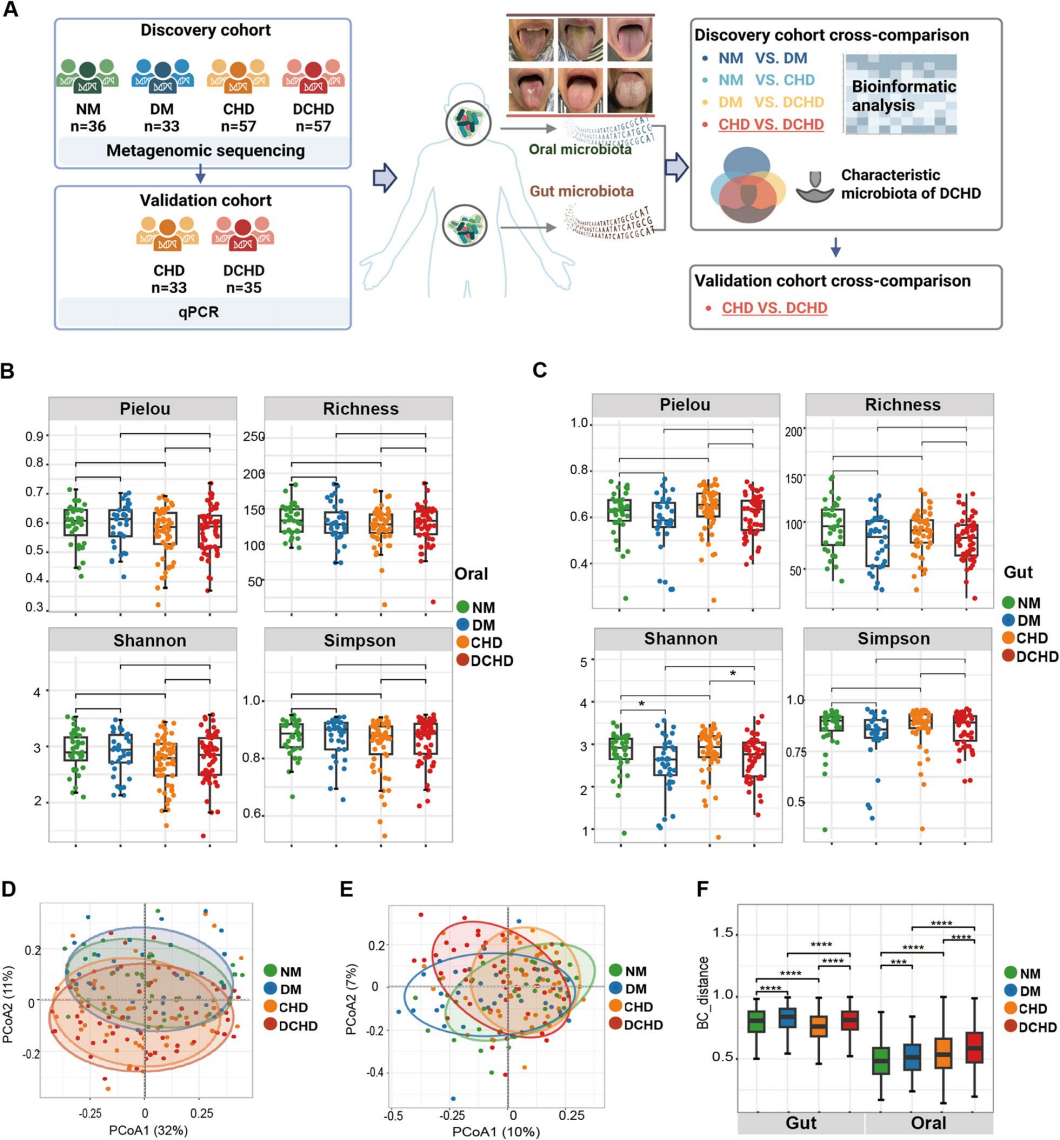
Alerted diversity of the oral and gut microbiota of participants with diabetic coronary heart disease (DCHD). **A** Flow chart of clinical cohorts. The discovery cohort focuses on the microbiota with differences between DCHD and coronary heart disease (CHD) and without differences between DM and NM, DCHD and DM, CHD and NM, which identify the characteristics of DCHD as an independent disease; microbiota with differences between DCHD vs. CHD, DCHD vs. DM, and CHD vs. NM but not DM vs. NM, which indicate the characteristics of microbiota in DCHD as a metabolic cardiovascular disease (DM may affect the microbiota characteristics of CHD). The validation cohort compares DCHD with CHD to validate the results of the discovery cohort. **B** α diversity including Pielou’s evenness, the Richness index, the Shannon index, and the Simpson index of oral microbiota in participants. **C** α diversity including Pielou’s evenness, the Richness index, the Shannon index, and the Simpson index of oral microbiota in participants. **D** PCoA of the oral microbiota in participants. E, PCoA of the oral microbiota in participants. **F** Bray–Curtis distance of the oral and gut microbiota in participants. *P < 0.05, ***P < 0.001

To investigate the association of the oral/gut microbiota with metabolic disturbance and DCHD and identify the characteristic microbiota of DCHD, we recruited 183 participants, of which, 36 had healthy controls (no DM or CHD), 33 had simple DM, 57 had simple CHD, and 57 had DCHD (Fig. 1A). The traditional cardiovascular risk factors and medication that may affect the microbiota [32, 33] are summarized in Table 1. Most of the items in the CHD group versus the DCHD group and the NM group versus the DM group were equal. We observed a significant increase in metformin use (*P* < 0.001), fasting blood glucose levels (FBG) (*P* < 0.001), and hemoglobin A1c (HbA1c) levels (*P* < 0.001) in CHD versus DCHD and NM versus DM. Regarding the expression of myocardial injury, cardiac troponin T (cTnT) levels significantly increased in the DCHD group compared with those in the CHD group (*P* < 0.05), however, the quartiles of cTnT in both groups were within the diagnostic threshold for myocardial injury. Total cholesterol (TC) and low-density lipoprotein cholesterol (LDL.C) levels significantly increased in the NM group compared with that in the DM group (*P* < 0.05), which may be due to more active use of statins in the DM group.

**Table 1:** Characteristics of the discovery cohort

	NM (N = 36)	DM (N = 33)	CHD (N = 57)	DCHD (N = 57)	*P* value
Age, year ^b^	59.28 ± 12.28	60.60 ± 12.94	63.44 ± 12.08	63.89 ± 10.57	*P* > 0.05
Male sex, n (%) ^c^	18(50.00%)	15 (45.45%)	43 (75.44%)	45 (78.95%)	† ‡
SBP, mmHg ^a^	137.50 (126.00, 149.00)	134.00 (122.50, 144.00)	142.00 (131.50, 151.50)	137.00 (127.00, 154.50)	*P* > 0.05
DBP, mmHg ^a^	85.00 (76.50, 93.00)	81.00 (77.00, 91.50)	79.00 (72.00, 85.50)	76.00 (69.50, 85.50)	‡
HR, bpm ^a^	78.50 (70.25, 87.00)	72.00 (66.50, 84.50)	71.00 (65.50, 76.00)	76.00 (66.00, 81.00)	*P* > 0.05
BMI, kg/m^2 a^	26.70 (24.77, 29.20)	26.64 (23.38, 28.47)	25.35 (23.33, 27.71)	25.35 (23.44, 27.59)	*P* > 0.05
Current smoke ^c^	10 (27.78%)	4 (12.12%)	16 (28.07%)	11 (19.30%)	*P* > 0.05
Smoking history ^c^	14 (38.89%)	8 (24.24%)	34 (59.65%)	39 (68.42%)	† ‡
alcohol consumption ^c^	8 (22.22%)	6 (18.18%)	25 (43.86%)	27 (47.37%)	† ‡
Medication					
Statins, n (%) ^c^	16 (44.44%)	18 (54.54%)	54 (94.74%)	48 (84.21%)	† ‡
Metformin, n (%) ^c^	0 (0%)	18 (54.54%)	0 (0%)	37 (64.91%)	* §
Laboratory data					
TG, mmol/L ^a^	1.51 (0.87, 2.75)	1.44 (0.89, 1.89)	1.09 (0.76, 1.60)	1.26 (0.95, 1.93)	*P* > 0.05
TC, mmol/L ^a^	4.87 (4.11, 5.54)	4.46 (3.65, 5.32)	3.53 (3.04, 4.13)	3.47 (2.99, 4.22)	* † ‡
HDL.C, mmol/L ^a^	1.15 (1.00, 1.28)	1.12 (0.91, 1.37)	1.03 (0.89, 1.23)	0.96 (0.81, 1.13)	‡
LDL.C, mmol/L ^a^	3.24 (2.40, 3.77)	2.73 (2.24, 3.44)	2.05 (1.51, 2.49)	1.93 (1.41, 2.60)	* † ‡
FBG, mmol/L ^a^	5.07 (4.75, 5.55)	7.06 (6.31, 8.24)	5.45 (5.00, 6.03)	6.61 (5.95, 7.61)	* §
HbA1c,% ^a^	5.80 (5.50, 5.88)	7.00 (6.65, 7.75)	6.00 (5.65, 6.20)	7.10 (6.50, 7.95)	* §
Scr, μmol/L ^a^	75.00 (65.25, 83.75)	69.00 (60.50, 78.00)	72.00 (66.00, 81.50)	79.00 (68.00, 88.00)	‡
BUN, mg/dL ^a^	14.98 (12.11, 17.22)	14.28 (10.22, 19.04)	14.28 (12.74, 16.66)	15.40 (11.76, 18.76)	*P* > 0.05
UA, μmol/L ^a^	323.50 (248.75, 371.75)	326.00 (263.50, 438.50)	330.00 (288.00, 384.00)	354.00 (319.50, 397.00)	*P* > 0.05
ALT, U/L ^a^	19.15 (14.15, 29.24)	16.00 (12.05, 27.15)	18.20 (12.45, 28.25)	17.10 (12.50, 25.15)	*P* > 0.05
AST, U/L ^a^	19.50 (16.03, 24.70)	18.80 (15.70, 21.70)	18.10 (15.15, 21.65)	16.60 (14.15, 21.70)	*P* > 0.05
cTnT, ng/mL ^a^	0.006 (0.006, 0.008)	0.009 (0.006, 0.010)	0.008 (0.007, 0.011)	0.011 (0.008, 0.015)	† ‡ §
NT-proBNP, pg/mL ^a^	41.37 (12.25, 65.50)	47.55 (16.39, 122.95)	107.70 (39.03, 198.15)	108.60 (44.14, 271.60)	† ‡

SBP: systolic blood pressure, DBP: diastolic blood pressure, HR: heart rate, BMI: body mass index, TG: triglyceride, TC: total cholesterol, HDL.C: high-density lipoprotein cholesterol, LDL.C: low-density lipoprotein cholesterol, FBG: fasting blood glucose, HbA1c: Hemoglobin A1c, Scr: serum creatinine, BUN: blood urea nitrogen, UA: uric acid, ALT: alanine transaminase, AST: aspartate transaminase, cTnT: cardiac troponin T, NT-proBNP: N-terminal pro-brain natriuretic peptide, NM: normal, DCHD: diabetic coronary heart disease

^*^. *P* < 0.05 for equality between NM and DM

^†^. *P* < 0.05 for equality between NM and CHD

^‡^. *P* < 0.05 for equality between DM and DCHD

^§^. *P* < 0.05 for equality between CHD and DCHD

*P* > 0.05 for no statistical differences among all groups

a. Median (IQR). b. Mean ± SD. c. n (%)

### Altered diversity of the oral and gut microbiota in DCHD

The diversity of oral and gut microbiota [34] demonstrates the microbial community characteristics under the condition of DCHD. The results showed that the α-diversity of oral microbiota was not significantly altered in healthy individuals, patients with diabetes only, patients with coronary artery disease only, and patients with DCHD (Fig. 1B), whereas the Shannon index of gut α-diversity was significantly lower in diabetic patients than in healthy individuals and in DCHD patients than in those with diabetes only (*P* < 0.05; Fig. 1C). This suggests that diabetes or coronary artery disease does not affect the richness and evenness of bacteria in the oral cavity, whereas hyperglycemia may reduce the unbalanced distribution of bacterial species in the gut.

The species differences in oral microbiota were small between the DCHD group and the coronary heart disease-only group (CHD group), while these two groups differed significantly from the diabetes-only group and the healthy group (Fig. 1D); the gut microbiota showed typical species differences between the DCHD group, the CHD group, and the diabetes-only group, distinctly differentiating the microbial communities of different patient groups (Fig. 1E). The Bray–Curtis distance also showed that the oral and gut microbiota of healthy individuals, patients with diabetes mellitus alone, patients with CHD alone, and patients with DCHD significantly differed (*P* < 0.001; Fig. 1F). DCHD and CHD typically differed in microbiota diversity, possessing two different bacterial community states.

### Changes in the composition of the oral and gut microbiota in patients with DCHD

**Fig. 2:**
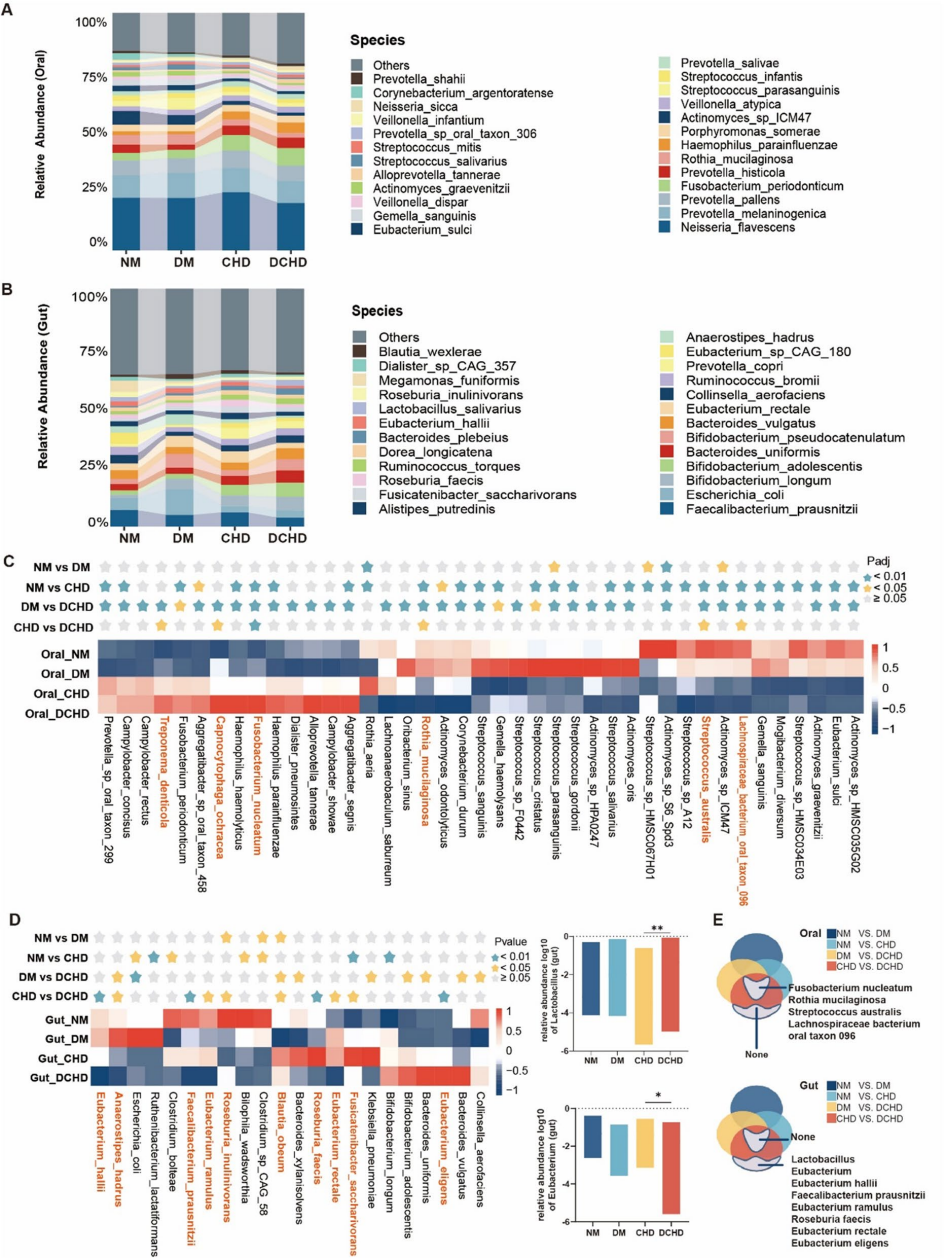
Compositional alterations of oral and gut microbiota in participants. **A** Stacked bar plots showing the relative abundances of oral microbiota at the species level in participants. **B** Stacked bar plots showing the relative abundances of gut microbiota at the species level in participants. **C** A star indicates the statistical difference of species and clustering heatmaps showing relative abundances of oral microbiota in participants. Statistically calculated using Padj. Species statistically different between CHD and diabetic coronary heart disease (DCHD) were marked orange. **D** A star indicates the statistical difference of species and clustering heatmaps showing relative abundances of gut microbiota in participants. Statistically calculated using the Pvalue. Species statistically different between CHD and DCHD were marked orange. Bar plots showing relative abundances of statistically significant genera in gut (*Lactobacillus* and *Eubacterium*). **E** Venn diagram summarizing the characteristic microbiota in DCHD (statistically different in CHD vs. DCHD, not statistically different in NM vs. DM, NM vs. CHD, and DM vs. DCHD; statistically different in CHD vs. DCHD, NM vs. CHD, DM vs. DCHD, not statistically different in NM vs. DM)

There were significant differences between the oral and gut microbiota at the phylum and species levels (Fig. 2A, B), with the oral microbiota being dominated by *Bacteroidetes, Proteobacteria, Firmicutes, Actinobacteria,* and *Fusobacteria,* and the gut microbiota being dominated by *Firmicutes, Bacteroidetes, Actinobacteria, Proteobacteria,* and *Verrucomicrobiade* (Additional file 1: Fig. S1A, B).

The gut microbiota differed between diseases (species level). The results showed that bacteria in the oral cavity have statistically significant differences exclusively in DCHD vs. CHD (Fig. 2C). *Fusobacterium nucleatum, Rothia mucilaginosa, Streptococcus australis,* and *Lachnospiraceae bacterium oral taxon 096* were positive in DCHD vs. CHD, DCHD vs. DM, and CHD vs. NM but negative in DM vs. NM (Fig. 2C). These species can serve as a distinctive microbial consortium associated with DCHD, and the presence of diabetes can further affect these microbiota (Fig. 2E). The abundance of *Fusobacterium nucleatum* increased in the oral cavity of patients with DCHD, while the abundance of *R. mucilaginosa, S. australis,* and *L. bacterium oral taxon 096* decreased (Fig. 2C).

We used the same approach to characterize the gut microbiota of DCHD and the results showed that *Eubacterium hallii, Faecalibacterium prausnitzii, Eubacterium ramulus, Roseburia faecis, Eubacterium rectale,* and *Eubacterium eligens* significantly differed exclusively in DCHD vs. CHD (i.e., no differences in DM vs. NM, CHD vs. NM, or DCHD vs. DM; Fig. 2D), and these species can be used as a DCHD-specific gut microbiota characteristic. We further screened the results at the genus level in order to identify highly specific gut microbiota. We found that *Lactobacillus* and *Eubacterium* significantly differed in patients with DCHD at the genus level (Fig. 2D). The guts of patients with DCHD exhibited an increased abundance of *Lactobacillus* and *E. eligens*, but a decreased abundance of *Eubacterium*, *E. hallii*, *F. prausnitzii*, *E. ramulus, R. faecis,* and *E. rectale* (Fig. 2E).

The comorbidity of diabetes and CHD presents a complex interplay with the microbial environment, where both diseases exert varying degrees of influence on the microbiota, and different anatomical sites (oral or gut) demonstrate distinct sensitivities to these disease states (Fig. 2C–E).In patients with CHD, regardless of diabetes status (NM vs. CHD and DM vs. DCHD), a diverse array of oral microbiota species was observed, the characteristic not mirrored in the gut microbiota. Conversely, in cases involving diabetes or CHD (CHD vs. DCHD or DM vs. DCHD), only a number of distinct species were detected in the gut microbiota.

### Association between the oral-gut microbiota and clinical parameters of cardiovascular-metabolic health

**Fig. 3:**
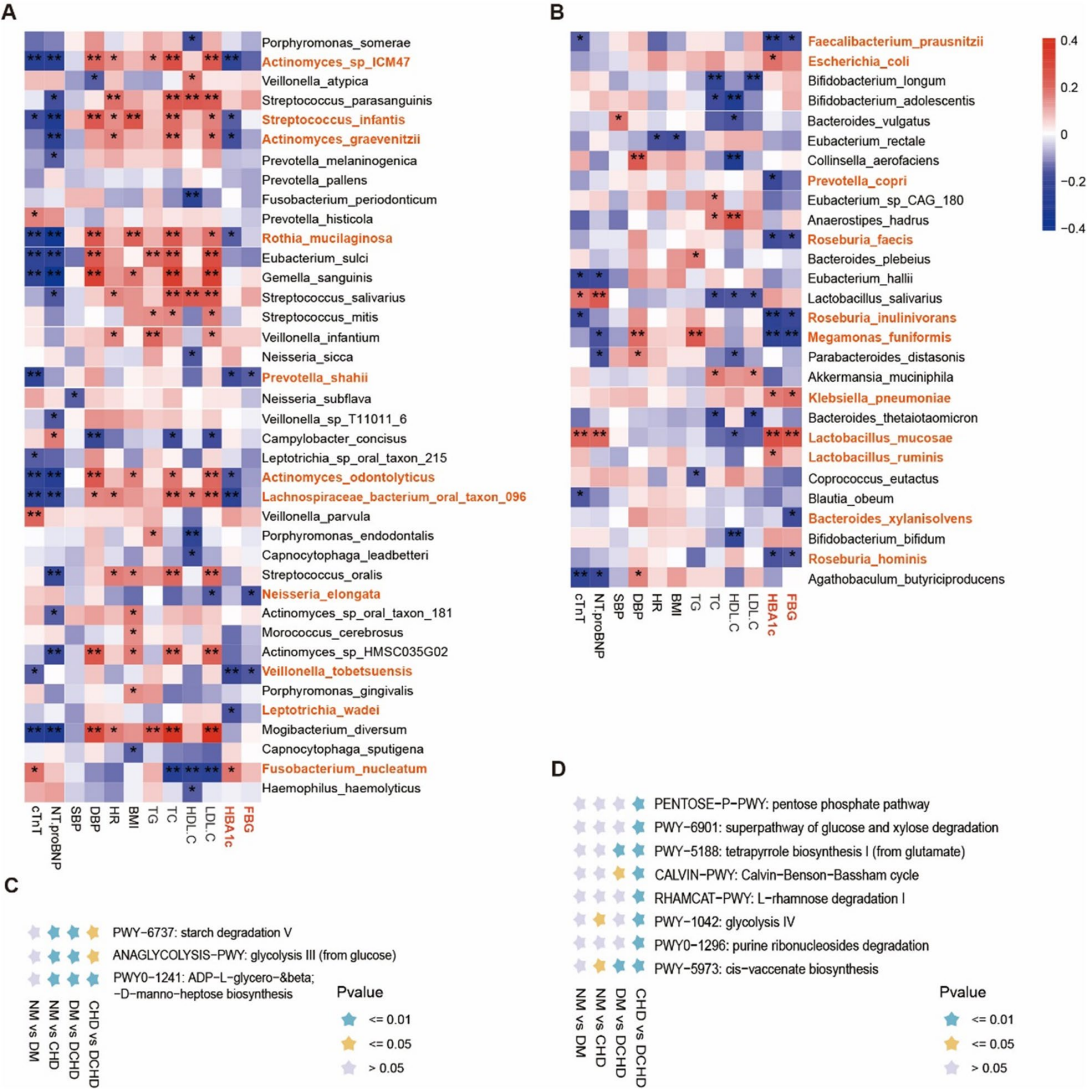
Associations between microbiota and the parameters related to cardiometabolic health. **A** and **B** Heatmaps of Spearman’s correlation coefficients between the clinical parameters and relative abundances of the top 50 species in the microbiota in participants. Species relevant to HbA1c or FBG were marked orange. **A**, oral **B**, gut. **C** and **D** Functional annotation of microbiota is statistically different between CHD and diabetic coronary heart disease (DCHD) based on the KEGG database. **C**, oral **D**, gut. cTnT: cardiac troponin T, NT-proBNP: N-terminal pro-brain natriuretic peptide, SBP: systolic blood pressure, DBP: diastolic blood pressure, HR: heart rate, BMI: body mass index, TG: triglyceride, TC: total cholesterol, HDL.C: high-density lipoprotein cholesterol, LDL.C: low-density lipoprotein cholesterol. *P < 0.05, ** P < 0.01

We demonstrated a strong relationship between the oral-gut microbiota and glycolipid metabolism and cardiac function through a Spearman correlation analysis. We hope to identify the characteristic microbiota of DCHD that is closely associated with glycolipid metabolism and is not affected by blood pressure and heart rate. HbA1c levels were positively correlated with oral *Fusobacterium nucleatum* abundance (*P* < 0.05; Fig. 3A), and negatively correlated with *Leptotrichia wadei*, *Veillonella tobetsuensis*, *Prevotella shahii*, *Actinomyces odontolyticus*, *L. bacterium oral taxon 096*, *Actinomyces graevenitzii*, *Streptococcus infantis*, *Actinomyces sp. ICM47*, and *R. mucilaginosa* abundance (*P* < 0.05; Fig. 3A). FBG levels were negatively correlated (*P* < 0.05) with oral *Neisseria elongata* and *P. shahii* abundance (Fig. 3A). HbA1c levels were positively correlated (*P* < 0.05) with the abundance of gut *Lactobacillus* (Additional file 1: Fig. S3A), as well as the abundance of gut *Escherichia coli*, *Klebsiella pneumoniae*, *Lactobacillus mucosae*, and *Lactobacillus ruminis* (Fig. 3B) but negatively correlated with the abundance of gut *Eubacterium* (Additional file 1: Fig. S3A) as well as the abundance of gut *F. prausnitzii*, *Prevotella copri*, *R. faecis*, *Roseburia inulinivorans*, *Megamonas funiformis*, and *Roseburia hominis* (*P* < 0.05; Fig. 3B). FBG levels were positively correlated (*P* < 0.05) with the abundance of gut *Lactobacillus* (Additional file 1: Fig. S3A) and gut *Klebsiella pneumoniae* and *L. mucosae* (Fig. 3B) but negatively correlated with the abundance of gut *Eubacterium* (Additional file 1: Fig. S3A) and gut *F. prausnitzii, R. faecis, R. inulinivorans, M. funiformis, Bacteroides xylanisolvens,* and *R. hominis* (*P* < 0.05; Fig. 3B). Thus, we found that oral *Fusobacterium nucleatum*, *L. bacterium oral taxon 096, R. mucilaginosa* and gut *Eubacterium***,** *F. prausnitzii*, *R. hominis*, and *R. faecis* not only serve as microbial markers of DCHD but are also closely related to blood glucose and may be involved in glucose metabolic homeostasis.

Functional annotation by Kyoto encyclopedia of genes and genomes (KEGG) [35] showed that the biological functions of oral microbiota were related to three glucose metabolic functions: ADP-L-glycero-β-D-manno-heptose biosynthesis, PWY-6737: starch degradation V, ANAGLYCOLYSIS-PWY: glycolysis III (from glucose) (Fig. 3C). The biological functions of gut microbiota were associated with eight other functions including PENTOSE-P-PWY: pentose phosphate pathway, PWY-6901: superpathway of glucose and xylose degradation, PWY-1042: glycolysis IV, RHAMCAT-PWY: L-rhamnose degradation, most of which were closely related to the metabolic degradation of sugars (Fig. 3D, Additional file 1: Fig. S2).

### Oral-gut microbial associations in DCHD

**Fig. 4:**
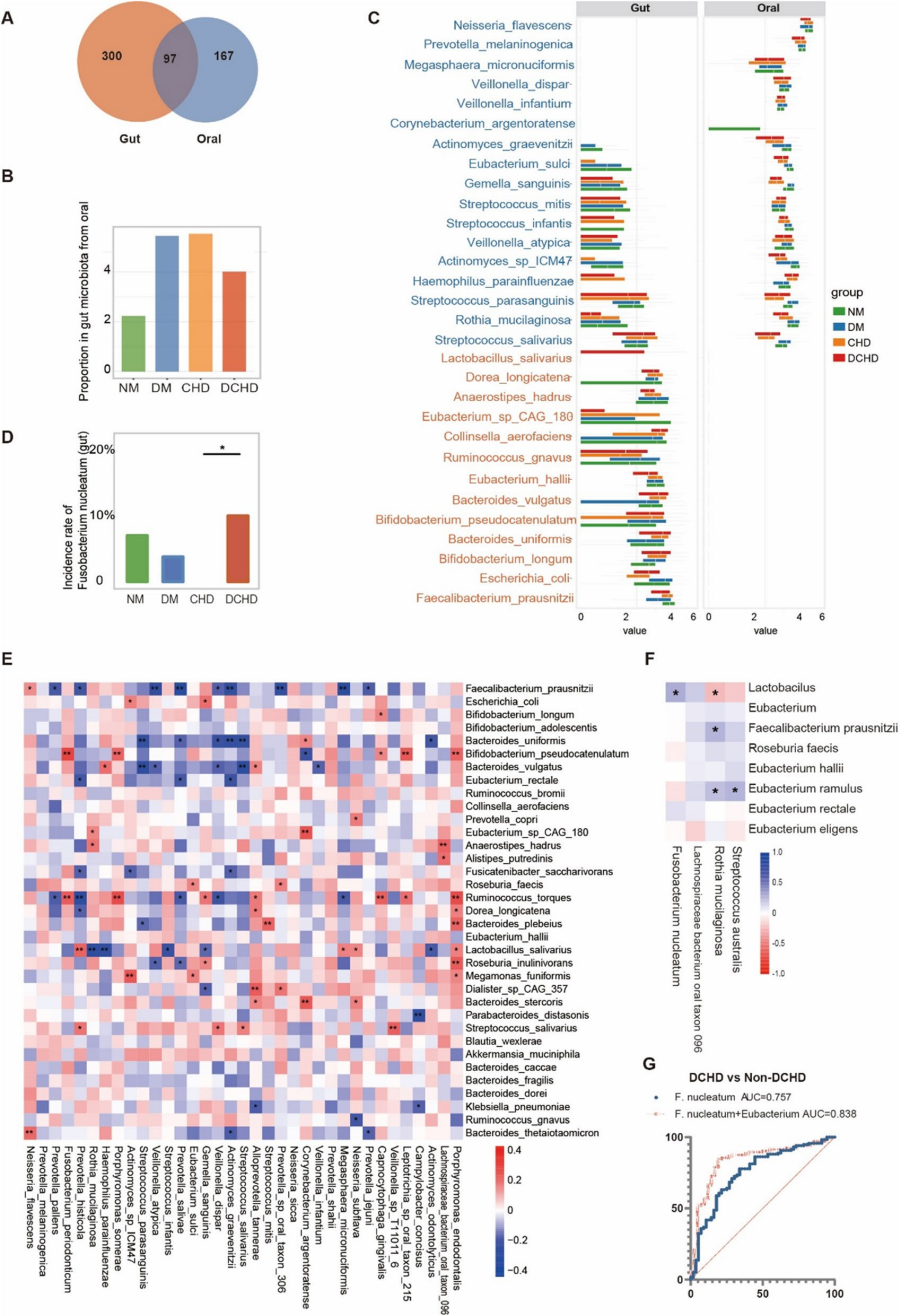
Characteristics of communications between the oral and gut microbiota in participants with diabetic coronary heart disease (DCHD). **A** Venn diagrams showing the unique and shared species between the oral and gut microbiota. **B** Mann–Whitney U test showing the proportions of the gut microbiota from the oral microbiota. **C** Relative abundances of the top 30 species shared by the oral and gut microbiota. Microbiota with higher relative abundances in the oral cavity than in the gut are colored blue (left), while microbiota with higher relative abundances in the gut are colored orange (left). **D** Chi-square analysis showing the prevalence of *Fusobacterium nucleatum* in the gut. **E** Heatmaps of Spearman’s correlation coefficients among relative abundances of the top 35 species within the oral cavity and the gut. N = 170 for all oral and gut samples corresponded. **F** Heatmaps of Spearman’s correlation coefficients among relative abundances of the “Characteristic microbiota of DCHD” in the CHD and DCHD groups. N = 102 for all oral and gut samples corresponded. **G** Combined diagnosis of oral-gut microbiota (*Fusobacterium nucleatum* and *Eubacterium*) to build a dependable diagnostic model based on the receiver operating characteristic. *P < 0.05, ** P < 0.01

Oral and gut microbiota are closely linked [36]. Whether oral-gut microbiota are closely related in DCHD, and whether oral-gut microbiota are involved in DCHD through ectopic colonization [37] or exacerbation [38] needs to be further explored. There are 97 common microbial species in both the oral cavity and gut, accounting for 36.74% of the total oral microbiota (Fig. 4A). Among the top 30 oral-gut shared bacteria, the abundance (Fig. 4C) and prevalence (Additional file 1: Fig. S3B) in the two sites differed greatly, with the same species of bacteria occupying different ecological niches in different colonization sites [39]. The fast expectation–maximization microbial source tracking (FEAST) [29] method was further used to examine the homology of the homologous bacteria. The results showed that there was no significant difference in the abundance of homologous oral-gut microbiota between different disease states (Fig. 4B). Disease states may influence the prevalence of microbiota, such as *F. nucleatum* (Fig. 4D). The abundance and prevalence of this bacterium was significantly higher in the oral cavity than in the gut, and disease did not significantly affect the abundance of *F. nucleatum* in the gut of patients with DCHD. However, DCHD increased the prevalence of the bacterium in the gut (Fig. 4D), indicating that the oral-gut distribution of specific bacteria is closely related to DCHD.

Several significant correlations were found between the top 35 abundant oral microbiota and the top 35 abundant gut microbiota (Fig. 4E). Focusing on the CHD and DCHD groups, the characteristic oral/gut bacteria of DCHD (Fig. 2E) were closely related, with oral *F. nucleatum* being positively correlated (*P* < 0.05) with gut *Lactobacillus*. *R. mucilaginosa* was positively correlated with gut *F. prausnitzii* and *E. ramulus* (*P* < 0.05) and negatively correlated with *Lactobacillus* (*P* < 0.05). Oral *S. australis* was positively correlated (*P* < 0.05) with gut *E. ramulus* (Fig. 4F). Meanwhile, the combined oral-gut microbiota abundance is more effective in the diagnosis of DCHD than oral or gut microbiota alone. The receiver operating characteristic (ROC) curves showed that a single DCHD-characteristic oral/gut microbiota could discriminate between DCHD and non-DCHD patients with a maximum discriminatory power of 0.756 (*Fusobacterium nucleatum*), whereas the combination of two indicators, the abundance of oral *Fusobacterium nucleatum* and the abundance of gut *Eubacterium intestinalis*, rapidly increased the area under curve (AUC) value to 0.838, achieving good diagnostic efficacy (Fig. 4G, Additional file 1: Fig. S4).

### Validation of the oral-gut microbiota in patients with DCHD

**Fig. 5:**
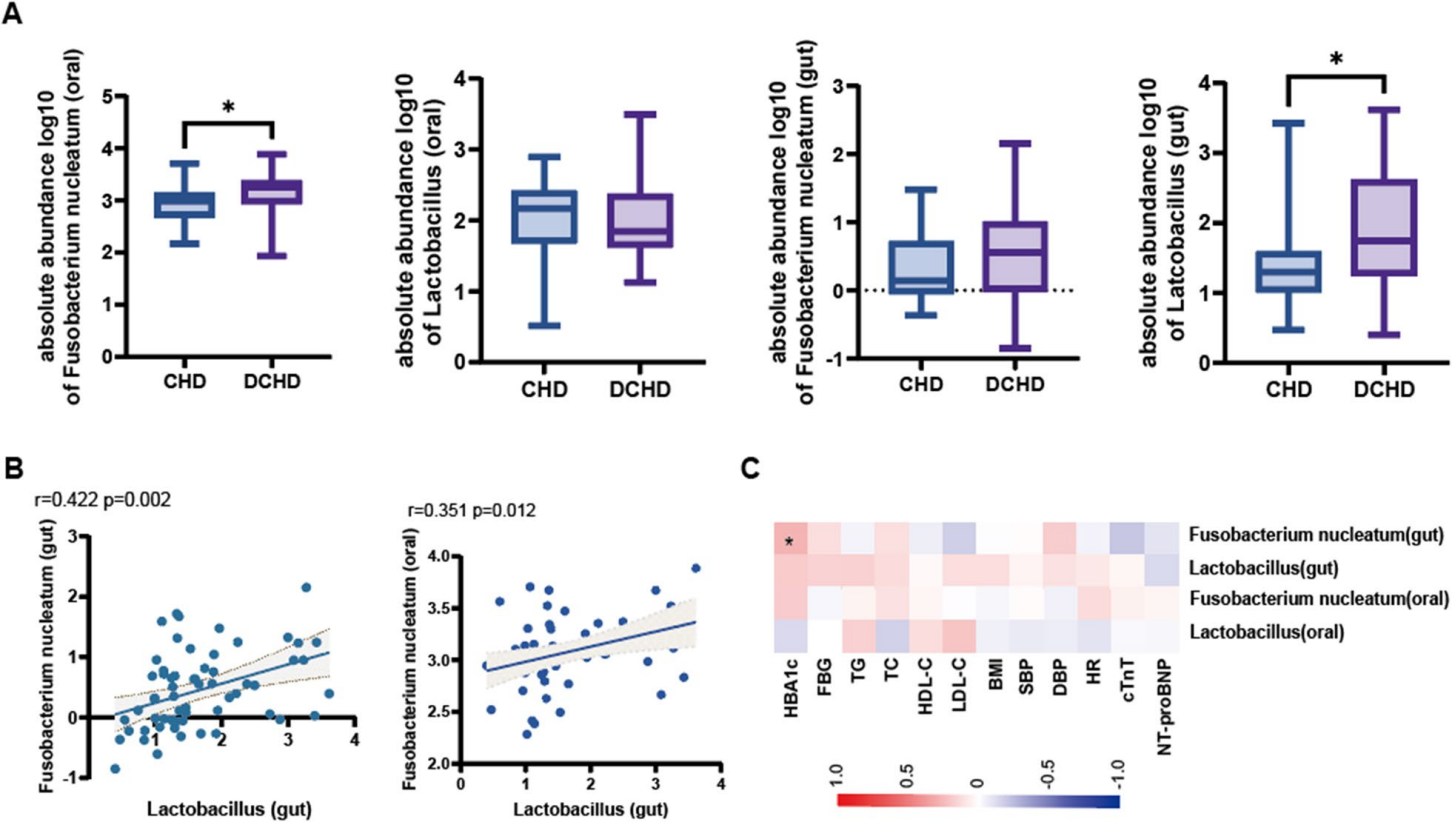
Validation of the oral-gut microbiota and the relationship with cardiometabolic health. **A** Absolute abundance of oral and gut *Fusobacterium nucleatum* and *Lactobacillus* in diabetic coronary heart disease (DCHD). **B** Simple liner regression of *Fusobacterium nucleatum* and *Lactobacillus*. **C** Heatmaps of Spearman’s correlation coefficients between absolute abundances of *Fusobacterium nucleatum*/*Lactobacillus* and clinical parameters. *P < 0.05

Metagenomic sequencing results may result in a high false-positive rate because an increase in one taxon in the constituent data is accompanied by a decrease in the others [40]. To confirm the utility of the metagenomic sequencing results, we set up a validation cohort, and the baseline conditions are shown in Table 2. There were no statistical differences in patient demographics, medication use, and relevant laboratory parameters. We performed qPCR quantification in the oral and gut samples for the four bacteria identified in the discovery cohort, including *F. nucleatum* and *Lactobacillus*, to understand the absolute quantification of the bacterial flora. The oral abundance of *F. nucleatum* in patients with DCHD was higher than that in patients with CHD (*P* < 0.05; Fig. 5A), and the gut abundance of *Lactobacillus* in patients with DCHD was higher than that in patients with CHD (*P* < 0.05; Fig. 5A), while the remaining bacteria did not statistically differ (Additional file 1: Fig. S5). Oral *F. nucleatum* had a positive correlation with gut *Lactobacillus* abundance (*P* < 0.05; Fig. 5B), and this correlation was retained in the DCHD group (*P* < 0.05) and disappeared in the CHD group (*P* > 0.05; Fig. S5). A correlation analysis of *F. nucleatum*, *Lactobacillus*, and clinical indicators of glycolipid metabolism indicated that the abundance of gut *F. nucleatum* was positively correlated with glycated HbA1c levels (*P* < 0.05). Together, these data indicated that oral *F. nucleatum*-gut *Lactobacillus* are a characteristic of the oral-gut microbiota axis of DCHD.

**Table 2:** Characteristics of the validation cohort

	DCHD (n = 35)	CHD (n = 33)	*P* value
Age, year ^b^	67.09 ± 10.78	65.97 ± 9.82	*P* > 0.05
Male sex, n (%) ^c^	24 (68.57%)	25 (75.76%)	*P* > 0.05
SBP, mmHg ^a^	144 (126, 157)	140 (131, 161)	*P* > 0.05
DBP, mmHg ^b^	82.29 ± 13.83	81.61 ± 11.63	*P* > 0.05
HR, bpm ^a^	76.0 (66.0, 86.0)	72.0 (64.5, 80.5)	*P* > 0.05
BMI, kg/m^2 b^	24.48 ± 3.08	24.51 ± 2.33	*P* > 0.05
Current smoke ^c^	9 (25.71%)	12 (36.36%)	*P* > 0.05
Smoking history ^c^	13 (37.14%)	17 (51.51%)	*P* > 0.05
alcohol consumption ^c^	7 (20.00%)	15 (45.45%)	*P* > 0.05
Medication			
Statins, n (%) ^c^	32 (91.43%)	32 (96.97%)	*P* > 0.05
Metformin, n (%) ^c^	20 (57.14%)	0 (0%)	*
Laboratory data			
TG, mmol/L ^a^	1.33 (0.88,1.94)	1.06 (0.82,1.60)	*P* > 0.05
TC, mmol/L ^a^	3.61 (2.88, 4.42)	3.32 (2.85, 3.95)	*P* > 0.05
HDL.C, mmol/L ^a^	0.91 (0.79,1.12)	0.95 (0.80,1.17)	*P* > 0.05
LDL.C, mmol/L ^a^	1.88 (1.43, 2.51)	1.73 (1.53,2.34)	*P* > 0.05
FBG, mmol/L ^a^	6.70 (5.49,9.25)	5.27 (4.94, 5.66)	*
HbA1c,% ^a^	7.20 (6.60, 8.10)	5.90 (5.50, 6.10)	*
Scr, μmol/L ^a^	71.00 (64.00, 82.00)	68.00 (58.00,81.50)	*P* > 0.05
BUN, mg/dL ^a^	16.52 (12.32, 18.48)	14.00 (12.36,18.76)	*P* > 0.05
UA, μmol/L ^a^	324.00 (293.00,359.00)	345.00 (271.00,425.00)	*P* > 0.05
ALT, U/L ^a^	18.30 (13.10, 24.10)	18.70 (13.05, 26.25)	*P* > 0.05
AST, U/L ^a^	18.90 (15.60, 22.70)	19.50 (17.00,21.65)	*P* > 0.05
cTnT, ng/mL ^a^	0.02 (0.01,0.02)	0.01 (0.01,0.01)	*P* > 0.05
NT-proBNP, pg/mL ^a^	62.56 (27.24, 146.40)	60.10 (26.49, 240.30)	*P* > 0.05

^*^. *P* < 0.05 for equality between diabetic coronary heart disease (DCHD) and CHD

a. Median (IQR). b. Mean ± SD. c. n (%)

### Diabetes promotes oral microbiota disruption and exacerbates myocardial ischemia–reperfusion injury

**Fig. 6:**
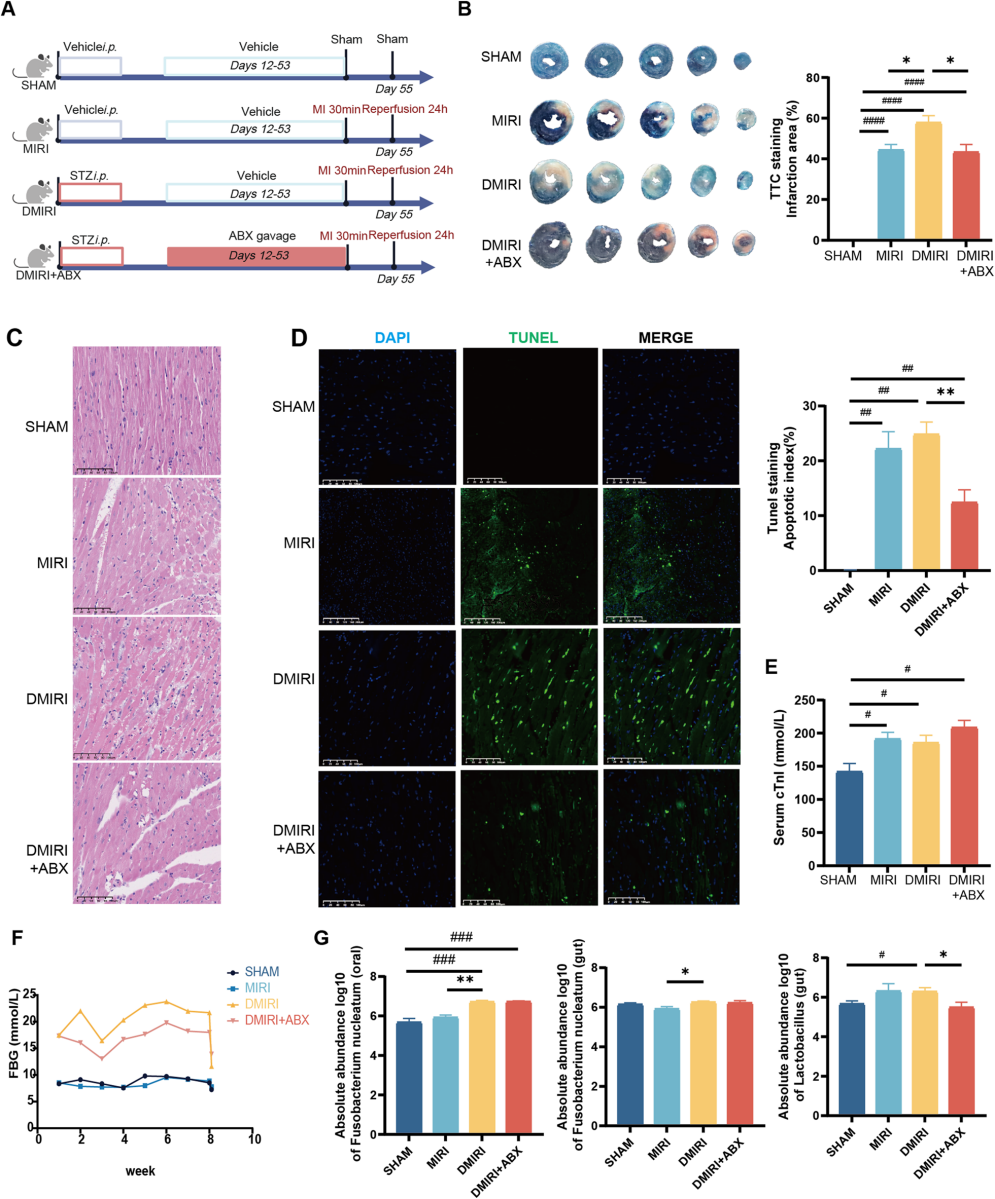
Hyperglycemia promotes microbiota disorder and aggravates myocardial ischemia–reperfusion injury. **A** Schematic illustration of experimental design. STZ: streptozotocin, ABX: antibiotic cocktail, MI: myocardial infarction (left anterior descending artery ligation). **B** TTC staining showing infarction area/left ventricular area (RA/LV) ratio in mice (N = 5). **C** Representative H&E staining of the left heart (Magnification × 20, N = 5). **D** Representative TUNEL staining of the left heart, bar plot showing apoptotic cells (green)/total number of cells (blue and green). (Magnification × 20, N = 5). **E** Serum cTnI levels (N = 4–6). **F** Fasting glucose change situation in mice (N = 9). The first record (W1) was at the first day after diabetic modeling and the last record was at the time of sampling. **G** Absolute abundance of *Fusobacterium nucleatum* and *Lactobacillus* in the oral cavity and gut (N = 5–7). SHAM: sham group, MIRI: myocardial ischemia-reperfusion injury group, DMIRI: diabetic myocardial ischemia-reperfusion injury group, DMIRI + ABX: pseudo-germ-free DMIRI group. Compared with the SHAM group, ^*#*^*P* < 0.05, ^*##*^*P* < 0.01, ^*###*^*P* < 0.001, ^*####*^*P* < 0.0001; * compared with the DMIRI group, *P < 0.05, **P < 0.01

To investigate whether diabetes affects oral-gut microbiota disorders and increases MIRI susceptibility, we first established a hyperglycemic mouse model using STZ intraperitoneally to confirm whether diabetes affected oral and gut microbiota (Fig. 6A). Evans blue/TTC staining showed that the myocardial infarct area increased in mice in the diabetic MIRI (DMIRI)group compared with mice in the MIRI group (*P* < 0.05), and the area of myocardial infarction was smaller in the DMIRI + ABX group than in the DMIRI group (*P* < 0.05; Fig. 6B). Hematoxylin and eosin (HE) staining showed that myocardial damage in the DMIRI group was more severe than that in the MIRI group, with significant disorganization of cardiomyocytes and inflammatory infiltration. Compared with the DMIRI group, the cardiomyocytes in the DMIRI + ABX group were more neatly arranged, and the degree of inflammatory infiltration was less severe (Fig. 6C). TUNEL staining indicated that there was no statistically significant difference in the apoptotic rate between mice in the DMIRI group and the MIRI group (P > 0.05), and that the apoptotic rate of mice in the DMIRI + ABX group decreased compared with that of the DMIRI group (*P* < 0.05), and the use of antibiotics could attenuate apoptosis at the early stage of DMIRI (Fig. 6D). Compared with the SHAM mice, serum cardiac troponin I (cTnI) levels increased in the MIRI and DMIRI groups (*P* < 0.05). The difference in serum cTnI levels between the MIRI, DMIRI, and DMIRI + ABX groups was not statistically significant (*P* > 0.05; Fig. 6E). Since there was no significant difference in the blood glucose levels between the DMIRI + ABX group and the DMIRI group throughout the whole process (Fig. 6F), regulating the microbiota by ABX may help reduce the cardiac susceptibility to MIRI in a chronic hyperglycemic state without a need to reduce the blood glucose level.

Mice were given antibiotics by gavage, followed by determining the abundance of oral and gut *F. nucleatum* and *Lactobacillus* in each group. There was no statistically significant difference in the abundance of oral and gut *F. nucleatum* and gut *Lactobacillus* in the DMIRI group vs. SHAM group (*P* > 0.05; Fig. 6G), and the MIRI event alone did not affect the oral and gut bacterial abundance. Mice in the DMIRI group exhibited an increase in the abundance of oral and gut *F. nucleatum* compared with mice in the MIRI group (*P* < 0.05), while no significant change in the abundance of gut *Lactobacillus was* observed (*P* > 0.05; Fig. 6G), indicating that the hyperglycemic state directly affected the oral and gut *F. nucleatum* abundance of mice. The oral and gut *F. nucleatum* abundance of mice in the DMIRI + ABX group was not significantly different from that of mice in the DMIRI group (*P* > 0.05), whereas gut *Lactobacillus* abundance decreased (*P* < 0.05); indicating that antibiotics regulated the abundance of gut *Lactobacillus*. Taken together, a hyperglycemic state directly affected the abundance of *F. nucleatum* but did not directly increase the abundance of gut *Lactobacillus*, while antibiotics reduced the abundance of *Lactobacillus* and also attenuated DMIRI. Regulation of microbiota is a protective measure for diabetic MIRI in addition to the regulation of blood glucose.

### F. nucleatum promotes oral-gut microbiota disruption and exacerbates myocardial ischemia–reperfusion injury

**Fig. 7:**
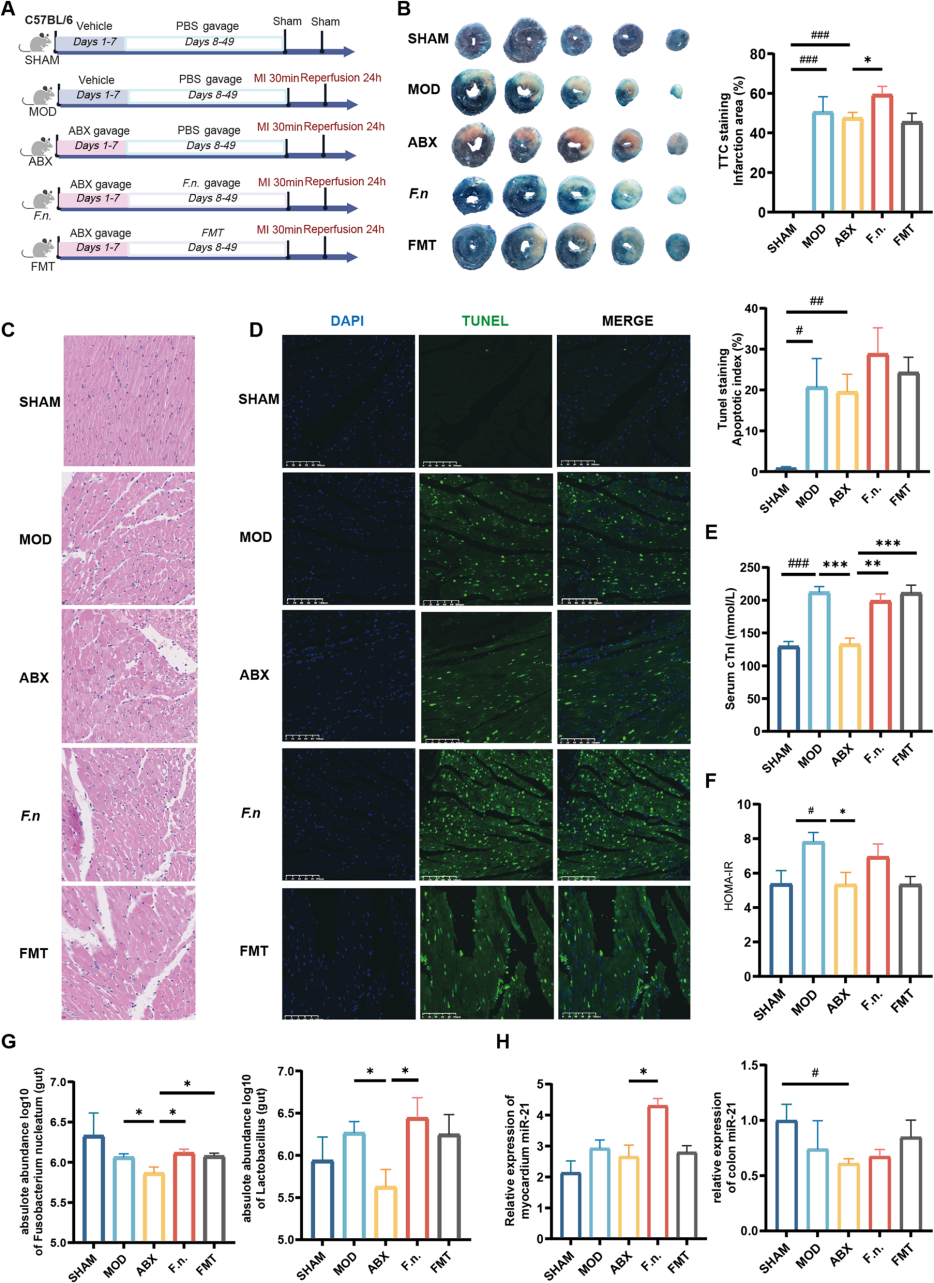
*Fusobacterium nucleatum* promotes oral-gut microbiota disorder and aggravates myocardial ischemia–reperfusion injury. **A** Schematic illustration of the experimental design. STZ: streptozotocin, ABX: antibiotic cocktail, MI: myocardial infarction (left anterior descending artery ligation), F.n.: *Fusobacterium nucleatum*, FMT: Fecal microbiota transplantation. **B** TTC staining showing infarction area/left ventricular area ratio in mice (N = 5). **C** Representative H&E staining of the left heart (Magnification × 20, N = 5). **D** Representative TUNEL staining of the left heart, bar plot showing apoptotic cells (green)/total number of cells (blue and green). (Magnification × 20, N = 4–5). **E** Serum cTnI levels (N = 5). **F** HOMA-IR index in mice (N = 4–5). **G** Absolute abundance of *Fusobacterium nucleatum* and *Lactobacillus* in the gut (N = 5–6). **H** Absolute abundance of miRNA-21 in the heart and colon (N = 3–4). SHAM: sham group, MIRI: myocardial ischemia–reperfusion injury group, DMIRI: diabetic myocardial ischemia–reperfusion injury group, DMIRI + ABX: pseudo-germ-free DMIRI group. Compared with the SHAM group, ^*#*^*P* < 0.05, ^*##*^*P* < 0.01, ^*###*^*P* < 0.001, ^*####*^*P* < 0.0001; * compared with the DMIRI group, *P < 0.05, **P < 0.01

Diabetes contributes to microbiota dysbiosis, but a causal relationship between oral *Fusobacterium nucleatum* affecting gut *Lactobacillus* and promoting MIRI is lacking. We performed *Fusobacterium nucleatum* transplantation in ABX-pretreated mice and fecal transplantation in the discovery cohort of patients with DCHD to confirm whether *Fusobacterium nucleatum* can exert the same pathogenic mechanism as “diabetic gut microbiota” (Fig. 7). Myocardial infarction area increased in the F.n. (*F. nucleatum gavage*) group compared with the ABX group (*P* < 0.05), while there was no significant difference in the FMT group compared with that in the ABX group (*P* > 0.05; Fig. 7B). Compared with the SHAM group, cardiomyocytes in the MOD and ABX groups were disorganized with unclear cellular boundaries, and inflammatory cellular infiltration could be observed in some areas (Fig. 7C). Compared with mice in the ABX group, mice in the F.n. and FMT groups exhibited increased disorganization of cardiomyocytes with nuclear pyknosis, lysis, and disappearance (Fig. 7C). There was no significant increase in the rate of apoptosis in mice in the F.n. group and the FMT group compared with that of mice in the ABX group (*P* > 0.05; Fig. 7D). While the serum cTnI level of mice in the F.n. and FMT groups increased compared with that of mice in the ABX group (*P* < 0.05; Fig. 7E). *Fusobacterium nucleatum* and the gut microbiota of patients with DCHD aggravated myocardial injury in mice, and the adverse effect of *Fusobacterium nucleatum* on MIRI was more significant.

The above MIRI process was accompanied by the regulation of oral/gut microbiota by *Fusobacterium nucleatum* and the gut microbiota of DCHD patients. The abundance of gut *Lactobacillus* and the abundance of gut *F. nucleatum* decreased in the ABX group of mice compared with the model (MOD) group of mice (*P* < 0.05; Fig. 7G). The gut *F. nucleatum* and *Lactobacillus* abundance in the F.n. group mice significantly increased (*P* < 0.05; Fig. 7G) compared with that in ABX group mice after administration of *F. nucleatum* solution. After administration of fecal bacterial fluids from DCHD patients, the abundance of gut *F. nucleatum* significantly increased in the FMT group of mice compared with that of the ABX group (*P* < 0.05; Fig. 7G), but the difference in the abundance of gut *Lactobacillus* was not statistically significant (*P* > 0.05; Fig. 7G). During this process, the insulin resistance level of mice in both the F.n. and FMT groups did not increase compared with that of the ABX group (*P* > 0.05; Fig. 7F). Therefore, *Fusobacterium nucleatum* might have exacerbated MIRI by affecting gut *Lactobacillus* in a pathway independent of blood glucose level, and the above effects on the gut microbiota were more pronounced than that of the overall gut microbiota of DCHD patients.

It has been suggested that *F. nucleatum* or *lactobacillus* may affect host miRNA-21 [41, 42]. Therefore, we examined the miRNA-21 in mouse colon and myocardium to confirm the association between the microbiota and miRNA-21. The results showed that myocardial miRNA-21 expression in mice in the F.n. group significantly increased compared with that in mice in the ABX group (*P* < 0.05; Fig. 7H), while no significant difference was observed in myocardial miRNA-21 expression in mice in the FMT group compared with that in mice in the ABX group (*P* > 0.05). The gut miRNA-21 expression in mice in the F.n. and FMT groups was also not significantly different from that in mice in the ABX group (*P* > 0.05; Fig. 7H). Therefore, the oral-gut microbiota dysbiosis caused by *F. nucleatum* might be associated with exacerbated MIRI, and the upregulation of myocardial miRNA-21 could potentially serve as a marker or potential target of myocardial injury.

## Discussion

Metabolic diseases such as diabetes contribute to the development and progression of CHD. The current consensus is that treatment of hyperglycemia should centrally revolve around the prevention and treatment of complications [43]. Human microbiome, functioning autonomously from glucose and lipid metabolic processes, plays a crucial role in the development and progression of metabolic cardiovascular disorders [44]. Elucidation of microbial-related mechanisms could reduce cardiovascular damage in diabetic patients and provide protection against the increasing prevalence of metabolic cardiovascular disease. Diseases associated with oral microbiota disorders, such as periodontitis, are risk factors for coronary heart disease [19, 45]. The oral-gut microbiota axis is among the mechanisms by which oral microbiota influences host disease [17]. In this paper, we explored for the first time the disease mechanism of DCHD through two clinical cohorts and two animal experiments, focusing on the characteristics and interrelationships of oral-gut microbiota.

Firstly, we analyzed the characteristics and functions of oral and gut microorganisms in patients with DCHD by metagenomic sequencing and verified the results using qPCR quantification. The simultaneous use of sequence analysis (relative abundance) and qPCR (absolute abundance) to quantitatively detect Fusobacterium nucleatum and Lactobacillus greatly increased the reliability of the data. We not only revealed the diversity and species differences of oral and gut microorganisms between DCHD and simple coronary artery disease but also uncovered the DCHD-specific bacterial characteristics by comparing healthy individuals to patients with diabetes mellitus alone. On this basis, we confirmed the correlation of DCHD-specific oral/gut microbiota with glycolipid metabolism, BMI, and cardiac function, and annotated the functions of the microbiota, thus confirming that these characteristic microbiota are not only the disease markers but also are closely associated with the cardiac metabolic function of the host, and are likely to be involved in the disease process of DCHD. We identified and validated oral *F. nucleatum* and gut *Lactobacillus* as characteristic oral/gut microbiota of DCHD. The discovery cohort showed that oral *Fusobacterium nucleatum*, *L. bacterium* oral taxon 096, *R. mucilaginosa*, gut *Eubacterium*, *F. prausnitzii*, and *Lactobacillus* can not only be used as microbial markers of DCHD but are also closely related to blood glucose. *Fusibacterium nucleatum* is related to oral mircobiota disorders [46], expressing proteins such as *Fusobacterium* apoptosis protein and *Fusobacterium* adhesin A [47], which may be involved in cardiovascular diseases [48]. Administering *Fusobacterium nucleatum* to a periodontitis mouse model can promote macrophage polarization and exacerbate atherosclerotic pathological changes in mice [49], suggesting that *Fusobacterium nucleatum* is a potential risk factor for patients with DCHD.

In this study, we found that “oral-gut microbiota correlation” also has significance both as a biomarker of DCHD and in participating in DCHD progression. DCHD is clinically common but complex, and its markers need to be both convenient and sensitive. Although oral or gut biomarker sets are currently available for the differential diagnosis of coronary heart disease and its subtypes [50], it is almost impossible to combine non-invasiveness (no blood collection) and simplicity. In this study, we found that the combination of oral and gut microbiota can be a simple, effective and non-invasive biomarker for DCHD. Meanwhile, oral microbiota is more likely to influence host disease by affecting the gut microecology than by ectopic colonization of the gut. Both ways contribute to the progression of the disease. In this study, the oral-gut microbiota did not exhibit high homology, and the homology even tended to decrease in patients with DCHD; however, a Spearman correlation analysis indicated a strong association between the oral-gut microbiota of different species, with the abundance of oral *Fusobacterium nucleatum* being positively correlated with the abundance of gut *Lactobacillus* in patients with DCHD and CHD. The correlation was validated in the validation cohort and was more significant in patients with DCHD.

Two animal experiments demonstrated that a hyperglycemic state increased oral *F. nucleatum* abundance. *Fusibacterium nucleatum* transplantation induced gut microbiota disruption, mainly characterized by increased gut *Lactobacillus* abundance, resulting in oral-gut microbiota disruption and the upregulation of myocardial miRNA-21 expression, exacerbating MIRI. In contrast, decreasing gut *Lactobacillus* abundance can alleviate DMIRI through a non-glucose-lowering pathway. Taken together, this study confirms the causal association of diabetes mellitus with oral-gut microbiota disruption and the consequent aggravation of MIRI. Microbiota regulate the disease through the host miRNA network [51], where *F. nucleatum* and *Lactobacillus* regulate host miRNA-21 [41, 42, 52]. Unfortunately, A limitation of this study is the lack of consideration for dietary differences that might influence research outcomes and did not explore the in-depth interaction mechanism between these two bacteria and the host miRNA-21.

The discovery of this oral-gut microbiota relationship is significant in that tight control of blood glucose levels in patients with type 2 diabetes mellitus does not completely reduce the incidence of macrovascular complications such as cardiovascular disease. Microbiota explains the insensitivity of some patients to glucose-lowering therapy or the mismatch between blood glucose reduction and cardiovascular benefits and provides more opportunities for treatment strategies and novel drug development for diabetic patients with coronary artery disease [13, 53]. At the same time, the oral microbiota in this study came from the tongue coating, which confirmed the scientific basis of tongue diagnosis and treatment of diseases in the TCM system. Diabetes can increase the bacterial load of the oral microbiota and alter the colonization of the gut by the patient’s oral microbiota [18]. Oral *Fusobacterium nucleatum* levels were significantly higher in diabetic patients and patients with coronary artery disease than in healthy individuals [54]. Antibodies to oral pathogenic bacteria such as *F. nucleatum* were detected in the sera of patients with coronary artery disease who were hospitalized for myocardial infarction [55]. Meanwhile, *Fusobacterium* influences the gut microbiota and is involved in Wnt/β-catenin signaling pathway [56]. Gut *Lactobacillus* may enter the bloodstream during acute myocardial ischemia and influence the severity of myocardial infarction [57]. Gut microbiota such as *Lactobacillus* may modulate myocardial adaptive immunity and participate in the process of myocardial injury through cellular metabolites [10, 57]. Although MIRI is an acute pathologic process, the diabetic state may increase the susceptibility to MIRI through long-term effects on the oral-gut microbiota. Oral-gut microbiota interactions, as represented by *F. nucleatum-Lactobacillus,* is a mechanism that deserves to be extensively explored in metabolic cardiovascular diseases.

## Conclusion

The adverse effects of diabetes mellitus on CHD are closely related to the oral-gut microbiota axis. Increased abundance of oral *Fusobacterium* nucleatum-gut *Lactobacillus* may not only serve as a microbiological signature of patients with DCHD but also as an intermediate in diabetes-exacerbated MIRI. The *F. nucleatum-Lactobacillus* axis exacerbates myocardial injury independently of the blood glucose. Targeting the oral-gut microbial axis is a potential strategy for the prevention and treatment of DCHD.

### Supplementary Information

**Additional file 1: Figure S1.** Composition of oral and gut microbiota at phylum and species level in discovery cohort. **A**. Stacked bar plots showing relative abundances of microbiota of tongue coating. **B**. Stacked bar plots showing relative abundances of microbiota of fecal. **Figure S2.** Functional modules of oral and gut microbiota in discovery cohort. **A**. Functional modules of oral microbiota. **B**. Functional modules of gut microbiota. Functional modules enriched in DCHD group are labeled yellow. **Figure S3.** Complementary analysis of oral-gut microbiota in discovery cohort. **A**. Correlation of* Lactobacillus* and *Eubacterium *in gut with clinical parameters. **B**. Oral and gut microbiota with greater variation between CHD and DCHD group. Select the median to calculate the result, Variation calculation method: (Δ relative abundance/relative abundance of CHD) ×100%. **C**. Prevalence of the top 30 species shared by the oral and gut. **Figure S4.** Predictive efficacy of oral/gut microbiota or the combination set of oral-gut microbiota for DCHD. AUC: area under the curve. **Figure S5.** Complementary analysis of oral-gut microbiota in validation cohort. **A**. Heatmap showing correlation between the species in oral and gut. **B**. Simple liner regression of oral *Fusobacterium nucleatum* and gut *Lactobacillus* in CHD and DCHD groups, respectively. **C**. Absolute abundance of oral and gut *Eubacterium, Eubacterium rectale *in DCHD. **Figure S6.** Abundance of *Fusobacterium nucleatum *(oral) and General condition in animal experiment II. **A**. Absolute abundance of *Fusobacterium nucleatum *(oral). **B**. Heart mass index. No statistical differences between groups.

**Additional file 2.** Method Details.

## Data Availability

The datasets used and/or analyzed for this study are available from the corresponding author upon reasonable request. This study did not generate unique code. Any additional information required to reanalyze the data reported in this paper is available from the Lead Contact upon request.

## Abbreviations

ABX: Antibiotic cocktail
ALT: Alanine transaminase
AST: Aspartate transaminase
AUC: Area under curve
BMI: Body mass index
BUN: Blood urea nitrogen
CHD: Coronary heart disease
cTnI: Cardiac troponin I
cTnT: Cardiac troponin T
DBP: Diastolic blood pressure
DCHD: Diabetic coronary heart disease
DM: Diabetes mellitus
DMIRI: Diabetic myocardial ischemia–reperfusion injury
FBG: Fasting blood glucose
FEAST: Fast expectation–maximization microbial source tracking
FMT: Fecal microbiota transplantation
F.n.: Fusobacterium nucleatum
HbA1c: Hemoglobin A1c
HDL.C: High-density lipoprotein cholesterol
HE: Hematoxylin and eosin
HR: Heart rate
KEGG: Kyoto encyclopedia of genes and genomes
LDL.C: Low-density lipoprotein cholesterol
MIRI: Myocardial ischemia–reperfusion injury
MOD: Model
NM: Normal
NT-proBNP: N-terminal pro-brain natriuretic peptide
ROC: Receiver operating characteristic
SBP: Systolic blood pressure
Scr: Serum creatinine
STZ: Streptozotocin
TC: Total cholesterol
TG: Triglyceride
TUNEL: Terminal deoxynucleotidyl transferase-mediated dUTP-biotin nick end labeling assay
