# Supplementary material for: Oral-gut microbial transmission promotes diabetic coronary heart disease
Source: Cardiovasc Diabetol. 2024 Apr 5;23:123. doi: 10.1186/s12933-024-02217-y (PMC10998415; doi:10.1186/s12933-024-02217-y)
Supplement: Supplementary file 1 — Additional file 1: Figure S1. Composition of oral and gut microbiota at phylum and species level in discovery cohort. A. Stacked bar plots showing relative abundances of microbiota of tongue coating. B. Stacked bar plots showing relative abundances of microbiota of fecal. Figure S2. Functional modules of oral and gut microbiota in discovery cohort. A. Functional modules of oral microbiota. B. Functional modules of gut microbiota. Functional modules enriched in DCHD group are labeled yellow. Figure S3. Complementary analysis of oral-gut microbiota in discovery cohort. A. Correlation of Lactobacillus and Eubacterium in gut with clinical parameters. B. Oral and gut microbiota with greater variation between CHD and DCHD group. Select the median to calculate the result, Variation calculation method: (Δ relative abundance/relative abundance of CHD) ×100%. C. Prevalence of the top 30 species shared by the oral and gut. Figure S4. Predictive efficacy of oral/gut microbiota or the combination set of oral-gut microbiota for DCHD. AUC: area under the curve. Figure S5. Complementary analysis of oral-gut microbiota in validation cohort. A. Heatmap showing correlation between the species in oral and gut. B. Simple liner regression of oral Fusobacterium nucleatum and gut Lactobacillus in CHD and DCHD groups, respectively. C. Absolute abundance of oral and gut Eubacterium, Eubacterium rectale in DCHD. Figure S6. Abundance of Fusobacterium nucleatum (oral) and General condition in animal experiment II. A. Absolute abundance of Fusobacterium nucleatum (oral). B. Heart mass index. No statistical differences between groups. [file 12933_2024_2217_MOESM1_ESM.docx]

**Additional files**

**Additional figures**


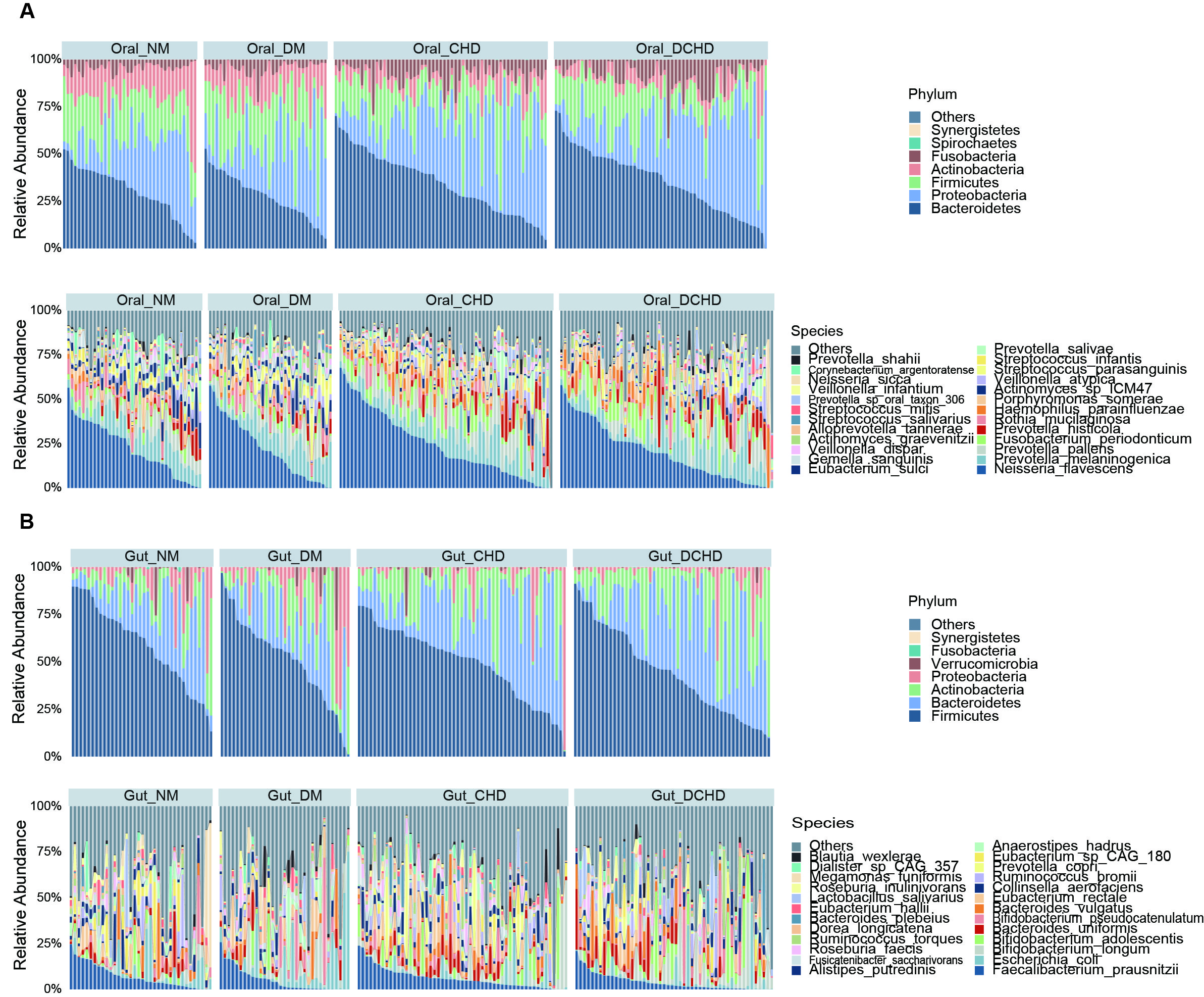


**Fig. S1** **Composition of oral and gut microbiota at phylum and species level in discovery cohort.** A. Stacked bar plots showing relative abundances of microbiota of tongue coating. B. Stacked bar plots showing relative abundances of microbiota of fecal.

**
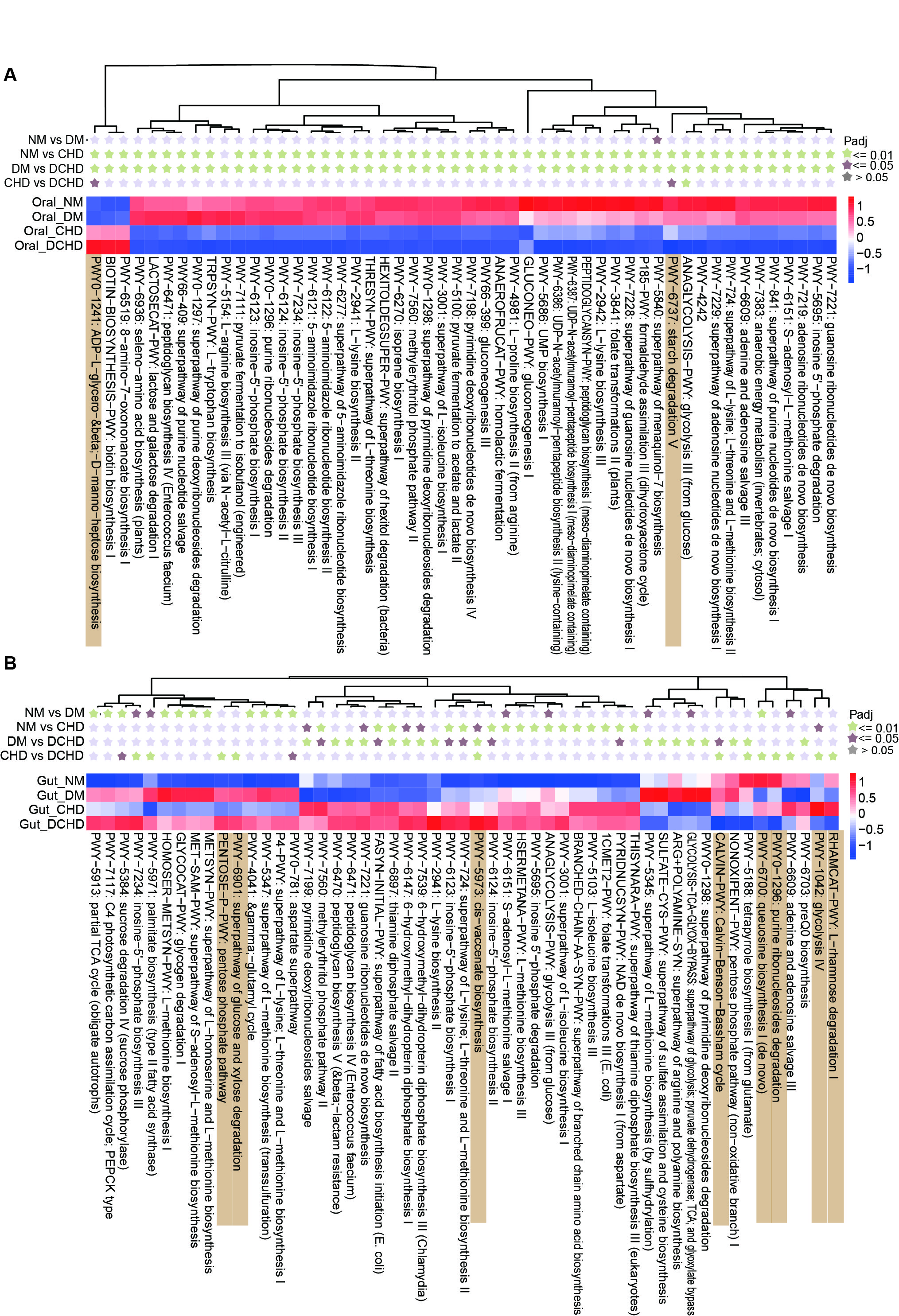
**

**Fig. S2** **Functional modules of oral and gut microbiota in discovery cohort.** A. Functional modules of oral microbiota. B. Functional modules of gut microbiota. Functional modules enriched in DCHD group are labeled yellow.


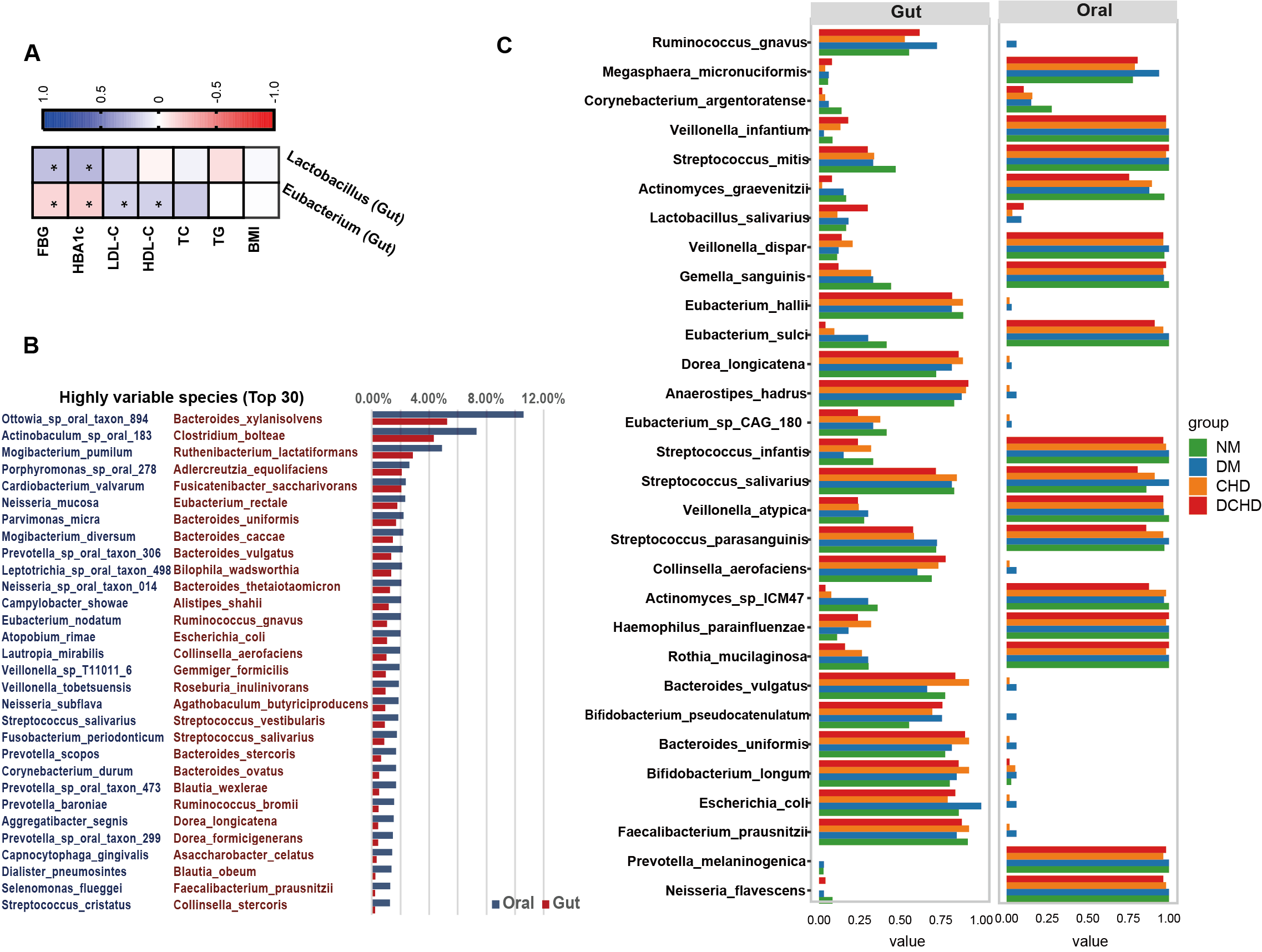


**Fig. S3** **Complementary analysis of oral-gut microbiota in discovery cohort.** A. Correlation of *Lactobacillus* and *Eubacterium* in gut with clinical parameters. B. Oral and gut microbiota with greater variation between CHD and DCHD group. Select the median to calculate the result, Variation calculation method: (Δ relative abundance/relative abundance of CHD) ×100%. C. Prevalence of the top 30 species shared by the oral and gut.


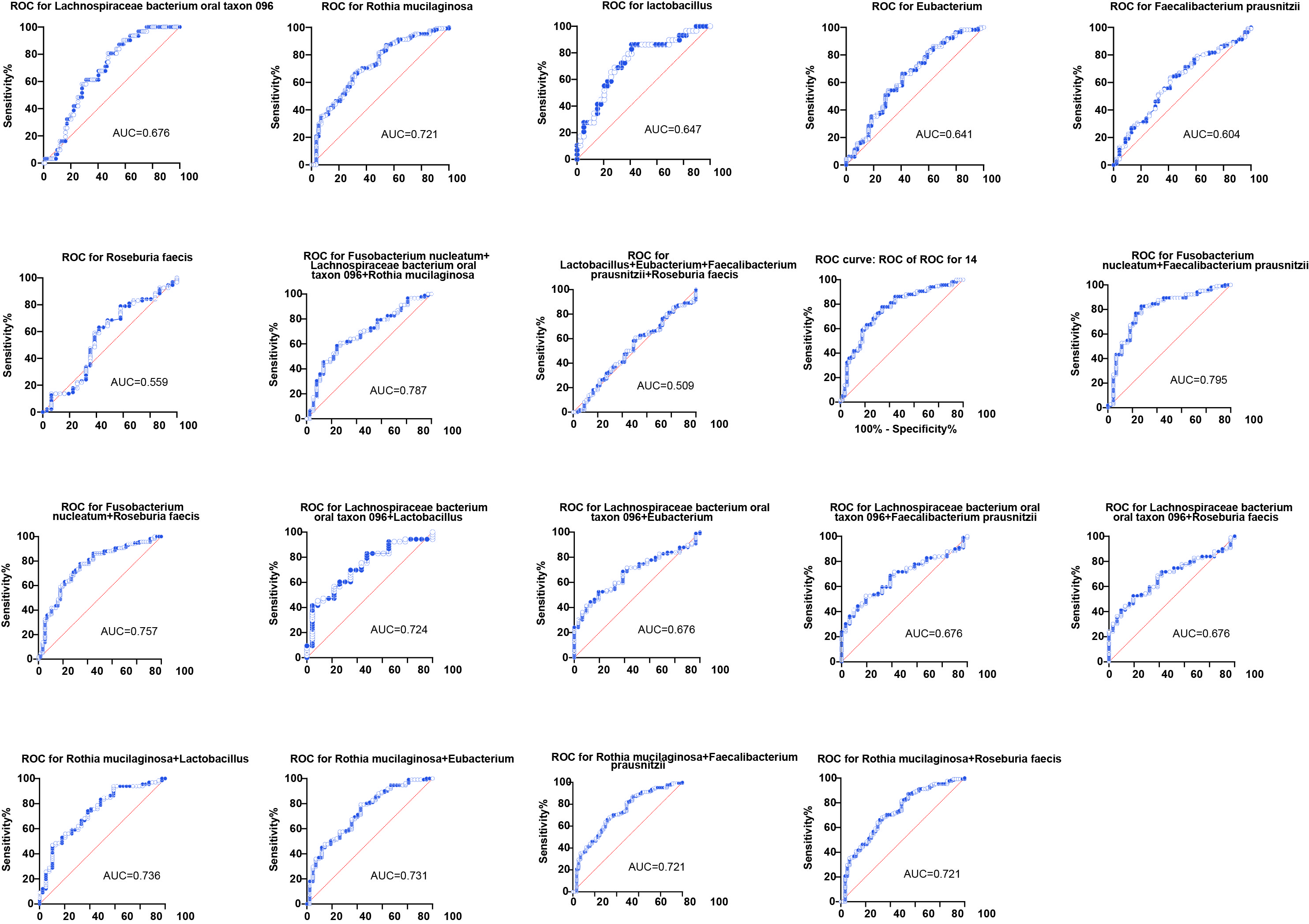


**Fig. S4 Predictive efficacy of oral/gut microbiota or the combination set of oral-gut microbiota for DCHD.** AUC: area under the curve.


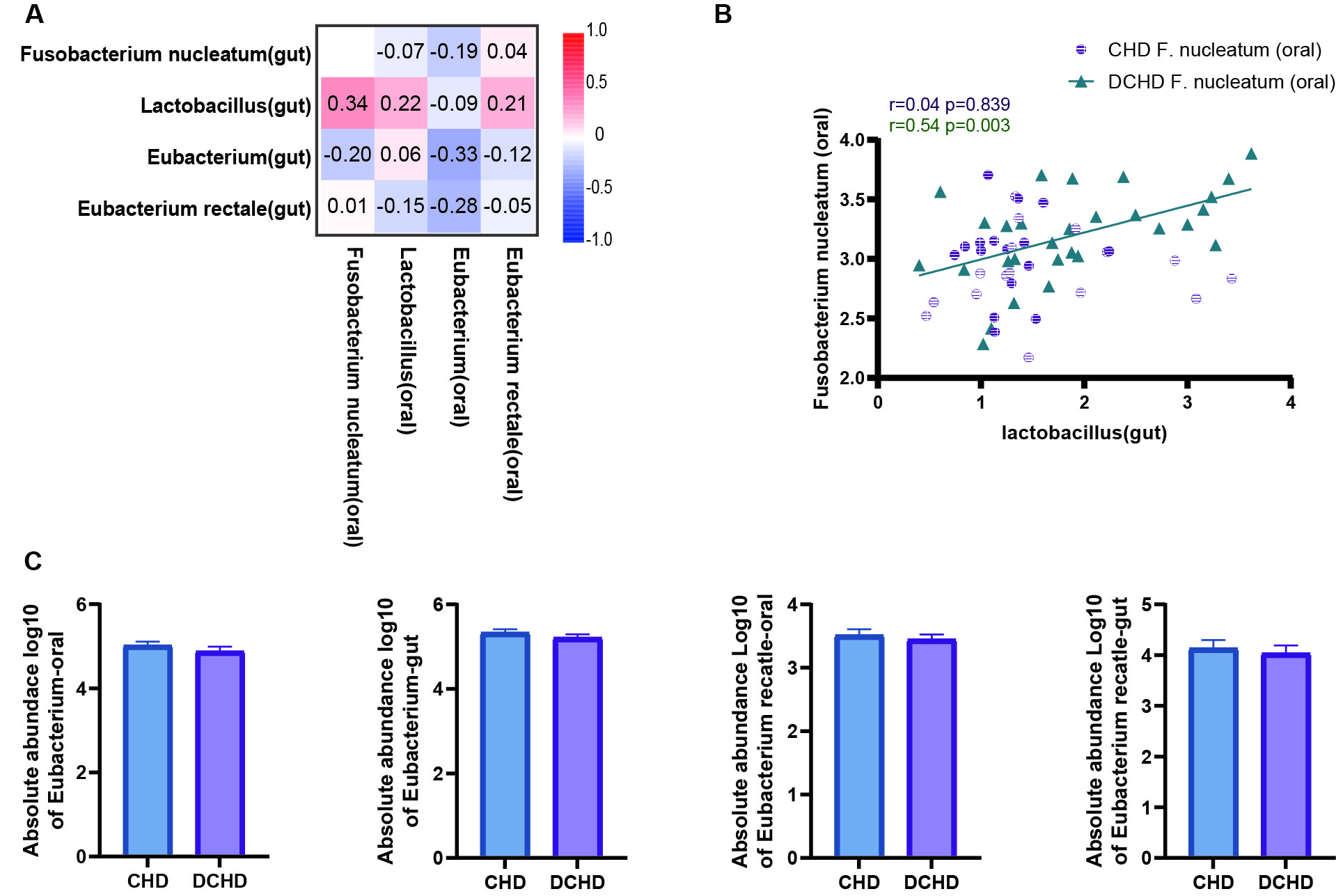


**Fig. S5 Complementary analysis of oral-gut microbiota in validation cohort.** A. Heatmap showing correlation between the species in oral and gut. B. Simple liner regression of oral *Fusobacterium nucleatum* and gut *Lactobacillus* in CHD and DCHD groups, respectively. C. Absolute abundance of oral and gut *Eubacterium, Eubacterium rectale* in DCHD.


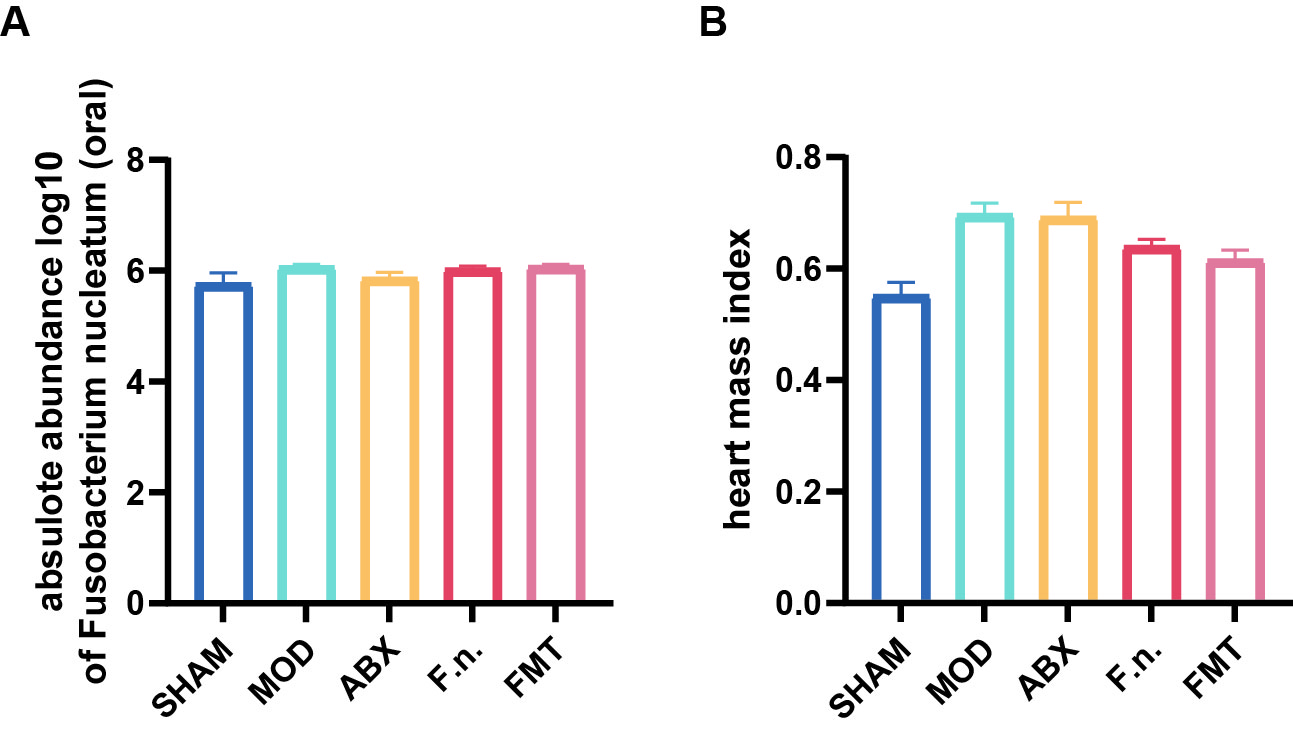


**Fig. S6 Abundance of *Fusobacterium nucleatum* (oral) and General condition in animal experiment II.** A. Absolute abundance of *Fusobacterium nucleatum* (oral). B. Heart mass index. No statistical differences between groups.
