# Supplementary material for: Oral-gut microbial transmission promotes diabetic coronary heart disease
Source: Cardiovasc Diabetol. 2024 Apr 5;23:123. doi: 10.1186/s12933-024-02217-y (PMC10998415; doi:10.1186/s12933-024-02217-y)
Supplement: Supplementary file 2 — Additional file 2. Method Details. [file 12933_2024_2217_MOESM2_ESM.docx]

**Additional files**

**Materials and methods**

**Sample size calculation**

Sample size was estimated using a previously published study sequenced oral-gut microbiota and the outcome were cardiovascular outcomes[1]. To achieve a minimum correlation coefficient of 0.25 (r=0.25), 5% significance level (α=0.05), and 80% test power (β=0.2) in correlation analyses, a sample size of 123 was required.

**Diagnosis Criteria**

Diabetes diagnosis criteria refer to the "Classification and Diagnosis of Diabetes: Standards of Medical Care in 2021"[2]：

1. Individuals with typical symptoms of diabetes and a glycated hemoglobin (HbA1c) level ≥ 6.5%.

2. Individuals with typical symptoms of diabetes and a fasting blood glucose level ≥ 7.0 mmol/L and/or a random blood glucose level ≥ 11.1 mmol/L.

3. Individuals with a history of diabetes and currently using antidiabetic medications.

The diagnosis of coronary heart disease follows the guidelines from the American College of Cardiology/American Heart Association Working Group on "Stable Ischemic Heart Disease Diagnosis and Management (2012)"[3]. Participants should conform to specific subtypes of stable coronary heart disease:

1. Coronary angiography indicates at least one coronary artery with >50% stenosis or has undergone coronary revascularization (including percutaneous coronary intervention and/or coronary artery bypass grafting).

2. History of previous acute coronary syndrome (ACS).[4, 5].

**Inclusion and exclusion criteria**

Participants with any of these conditions were excluded: 1. Acute myocardial infarction, congenital heart disease, severe cardiac dysfunction (NYHA class ≥Ⅲ), uncontrolled severe hypertension (systolic blood pressure ≥180 mmHg and/or diastolic blood pressure ≥110 mmHg), Patients with grade Ⅱ or above type Ⅱ sinus or atrioventricular block without pacemaker implantation, difficult to control malignant arrhythmia, pulmonary embolism, severe valvular disease, etc; 2. lack of teeth (less than 20 teeth) or associated with severe oral diseases, chronic diarrhea or severe constipation of the digestive tract;3.patients with severe respiratory system, nervous system, hematological system, autoimmune diseases and malignant tumors; 4. severe liver and kidney dysfunction (alanine aminotransferase and/or aspartate aminotransferase 3 times higher than normal, creatinine higher than normal); 5. patients with severe trauma, infection or surgical operation history in the past month;6. use of antibiotics and probiotic products within 3 months; 7. those who were participating in other clinical trials.

**Phenotyping and metadata collection**

The clinical data collected in this study encompass comprehensive demographic information, medical history, history of diabetes and statin use, and severity of coronary artery atherosclerotic lesions. Resting heart rate and blood pressure were measured, with blood pressure obtained from the left side while the subject was in a sitting position. Smoking history was defined as smoking ≥1 cigarette per day, continuously or cumulatively for more than 6 months; recent smoking history was defined as an average of ≥1 cigarette per day over the past 3 months. Alcohol history was defined as consuming >50 g of alcohol per day.

Blood Sample Collection: Peripheral venous blood was drawn in the morning on an empty stomach. The measured parameters included complete Systolic Blood Pressure (SBP), Diastolic Blood Pressure (DBP), triglycerides(TG), cholesterol (TC), high-density lipoprotein cholesterol (HDL-C), low-density lipoprotein cholesterol (LDL-C),alanine aminotransferase (ALT), aspartate aminotransferase (AST), creatinine (Cr), blood urea nitrogen (BUN), uric acid (UA), fasting blood glucose (FBG), glycated hemoglobin (HBA1c), creatine kinase-MB (cTnT), N-terminal pro-B-type natriuretic peptide (NTproBNP), etc. After blood collection, the samples were immediately sent to the laboratory for analysis.

Oral Microbiota Collection[6]: Use a swab to collect a sample of the patient's tongue coating. In the morning, before the patient eats or drinks, scrape the swab from the base to the tip of the tongue, rolling the swab 30 times. Place the swab into an EP tube containing 1 mL of phosphate-buffered saline (PBS), gently agitate to elute the tongue coating microbiota from the swab. Repeat the process with a new swab two more times to ensure thorough collection of the tongue coating sample. After collection, centrifuge (10000×g, 4 ℃, 15 min), gently absorb and discard the supernatant, and retain the precipitate as the sample. Freeze rapidly in liquid nitrogen and store at -80 ℃ until analysis.

Intestinal Microbiota Collection [7]: Use a sterile fecal collector to collect the sample. The subject will excrete feces into a clean container, taking care to avoid contamination from urine, toilet bowl surfaces, etc. Collect the sample immediately after defecation, preferably obtaining feces from the posterior part, and trying to collect feces from the inner side of the middle part. Place the fecal sample into a sterile EP tube. Freeze the sample in liquid nitrogen within 2 hours of collection and store at -80 ℃ until analysis.

**DNA library construction and sequencing**

The genomic DNA was randomly sheared into short fragments. The obtained fragments were end repaired, A-tailed and further ligated with Illumina adapter. The fragments with adapters were PCR amplified, size selected, and purified. The library was checked with Qubit and real-time PCR for quantification and bioanalyzer for size distribution detection. Quantified libraries will be pooled and sequenced on Illumina platforms, according to effective library concentration and data amount required.

**Metagenome sequencing and analysis**

Low-quality raw paired-end reads and adapters were removed using trim-galore v0.5.0 (q > 20) and cut-adapter v1.11 (PMID: 28715235). Filtered high-quality reads were aligned to the human genome assembly (hg38) using Bowtie2 v2.1.0 (PMID: 22388286) for de-human contamination. The unaligned paired-end reads were merged, based on the ChocoPhlAn database, species composition annotation was performed using MetaPhlAn3 to obtain species abundance tables. Functional annotation was performed using HUMANN3 to obtain the MetaCyc pathway[8] and Gene family abundance table, and the KEGG abundance table was obtained based on the Gene family abundance table using the humann regroup table command. From the abundance table at each taxonomic level, the number of annotated genes was counted and the functional comparison between groups was performed.

Metabolic pathways were tested using Mann-Whitney U test and visualized by corrplot R package. Correlations between species were analyzed using Spearman correlation. When multiple comparisons were performed, Benja mini-Hochberg corrected p-values were used.

**qPCR sequencing primer sequence in validation cohort**

**Table1. Primer design**

| **Full name of primers** | **Primer sequence（5' to 3'）** | |
| --- | --- | --- |
| *Fusobacterium nucleatum* | upstream primer | CAACCATTACTTTAACTCTACCATGTTCA |
| *Fusobacterium nucleatum* | downstream primer | GTTGACTTTACAGAAGGAGATTATGTAAAAATC |
| *Latcobacillus* | upstream primer | GAGGCAGCAGTAGGGAATCTTC |
| *Latcobacillus* | downstream primer | GGCCAGTTACTACCTCTATCCTTCTTC |
| *Eubacterium* | upstream primer | CGGTACCTGACTAAGAAGC |
| *Eubacterium* | downstream primer | AGTTTCATTCTTGCGAACG |
| *Eubacterium rectale* | upstream primer | CATTGCTTCTCGGTGCCGTC |
| *Eubacterium rectale* | downstream primer | ATTTGCTCGGCTTCACAGCT |

**Animal modeling**

**Diabetes Modeling**

C57BL/6J mice, maintained on a standard diet, were induced with diabetes by intraperitoneal injection of 50 mg/kg streptozotocin (STZ) for five consecutive days. Fasting blood glucose levels were monitored continuously for one week after induction. A fasting blood glucose level exceeding 200 mg/dL within the first week confirmed the successful establishment of the diabetes model[9, 10].

**Pseudo-sterile Modeling**

Broad-spectrum Antibiotic (ABX) Regimen: A solution containing ampicillin (1 g/L) (Shanghai Aladdin Biochemical Technology Co.,Ltd., China (A105483)), neomycin sulfate (1 g/L) (Sigma-Aldrich, USA (N6386)), metronidazole (1 g/L) (Sigma-Aldrich, USA (M1547)), and vancomycin (0.5 g/L) (Shanghai Aladdin Biochemical Technology Co.,Ltd., China (V105495)) was prepared. The solution was administered once daily via gavage for a duration of six weeks, prepared freshly each day[11].

**Preparation of Human Fecal Microbiota Suspension**

Fecal samples from 10 clinical patients* (≥1 g/sample) were combined. After mixing, the fecal material was resuspended in phosphate-buffered saline (PBS) at a 1:4 ratio and homogenized. The fecal suspension was centrifuged at 6000 g, 4 ℃ for 15 minutes, and the collected supernatant was considered the fecal microbiota suspension ready for transplantation.

For randomization, fecal samples from the first part of the clinical study, specifically from DCHD patients (n=10), were combined to create a fecal mixture for Fecal Microbiota Transplantation (FMT). The inclusion and exclusion criteria for human donors, as well as the fecal collection method, were consistent with those in the discovery cohort.

**Table 2. Characteristics of feacl microbiota transplantation donors.**

|  | **Feacl microbiota transplantation donors N=10** |
| --- | --- |
| Age, yrs ^b^ | 66.5±10.85 |
| Gender, n (M/F) ^c^ | 70%(7) |
| SBP, mmHg ^b^ | 139.20±17.65 |
| DBP, mmHg ^b^ | 82.40±17.28 |
| Heart rate, bpm ^b^ | 80.20±17.12 |
| BMI, kg/m^2 b^ | 23.43±2.80 |
| Current smoke ^c^ | 30%(3) |
| Smoking history ^c^ | 40%(4) |
| Statins, %(n) ^c^ | 90%(9) |
| Metformin, %(n) ^c^ | 70%(7) |
| TG, mmol/L ^a^ | 3.99 (2.975, 4.485) |
| TC, mmol/L ^a^ | 1.93 (0.948, 3.855) |
| HDL-C, mmol/L ^a^ | 0.885 (0.735, 0.9225) |
| LDL-C，mmol/L ^a^ | 2.33 (0.97, 2.685) |
| FBG，mmol/L ^a^ | 8.12 (4.875, 9.365) |
| HBA1c，% ^a^ | 7 (6.075, 8.825) |

a. Median (IQR). b. Mean SD. c. n (%)

**Myocardial Ischemia-Reperfusion Injury (MIRI) Modeling**

Preoperative: Weigh and record the weight of all animals. Prepare animals for surgery by shaving the fur, induce anesthesia using isoflurane, and position the animals on a small animal surgical table in a supine position with limbs secured using 3M tape after the animals exhibit anesthesia responses such as disappearing corneal reflex and decreased muscle strength. After ensuring deep anesthesia, install the electrocardiogram monitor. Intraoperative: Disinfect the local skin, make an incision between the ribs after draping, bluntly dissect the muscles, open the pericardium, and expose the heart. Extend the heart outside the body, exposing the left anterior descending coronary artery. Ligature is applied approximately 1-2 mm from the bottom edge of the left atrial appendage using a 6-0 sterile silk thread. After confirming a whitening of the heart base, promptly close the chest, squeeze to prevent pneumothorax, and suture the muscles and skin layer by layer, inducing ischemia in the anterior wall of the left ventricle. After 30 minutes of ischemia, a second thoracotomy is performed, the ligature is released to restore blood flow to the left anterior descending coronary artery, and the chest is promptly closed and sutured to prevent pneumothorax.

**Specimen Collection**

Oral Microbiota Collection of mice[1]: Fix the mouse, gently scrape the entire oral cavity (tongue, sublingual, gingiva, and saliva) with swab, cut the swab head with sterile scissors, place it in a sterile cryogenic tube, and immediately transfer it to -80 ℃ after rapid freezing in liquid nitrogen.

Intestinal Content Collection of mice[1]: Open the mouse abdomen to find the location of the ileocecal valve, collect the terminal ileum and below intestinal tissues, squeeze intestinal contents into a sterile cryogenic tube using sterile forceps at the terminal ileum, and immediately transfer it to -80 ℃ after rapid freezing in liquid nitrogen.

**Detection Methods**

**TTC-Evans Blue Staining**

Heart slices were immersed in 1% 2,3,5-Triphenyl-2H-tetrazolium chloride (TTC) (Sigma-Aldrich, USA (T8877-50G)) in the dark. Blue areas represent the non-ischemic zone, red areas indicate the myocardial ischemic at-risk zone, and white areas indicate the myocardial infarction zone. The fixed tissue was photographed using a digital camera, and Image-ProPlus 6.0 software (National Institutes of Health, Bethesda, USA) was used for assessment, calculating the myocardial infarction area (%).

**Hematoxylin-Eosin（HE）Staining**

Collection of cardiac samples with a thickness of 3 mm, fixed with 4% paraformaldehyde and embedded in paraffin. Cross sections were stained with hematoxylin and eosin (Boruijie Technology Co., Ltd., China (DH0001/0405A17), Beijing Chemical Plant, China (20150915)). All images were captured using Leica DMi8 microscope.

**Tunel Staining**

The heart samples were separated and fixed in 10% phosphate-buffered formalin for 24 h, subsequently embedded in paraffin, sliced (4–5 μm). Terminal deoxynucleotidyl transferase-mediated dexoxyuridine triphosphate nick-end labeling (TUNEL) staining was performed using the TUNEL BrightGreen Apoptosis Detection Kit (Roche, Swiss) following manufacturer’s instructions. Apoptotic nuclei were labeled with green fluorescein staining and total cardiomyocyte nuclei were marked with DAPI. The pictures of heart tissues were viewed by confocal microscopy. Rate of apoptosis was displayed as ratio of TUNEL positive nuclei to DAPI-stained nuclei.

**Enzyme-linked immunosorbent assay (ELISA) analysis**

After treatment, blood samples were collected. Serum levels of cardiac troponin I (cTnI) (mm-0791M1) and insulin (mm-0579M1) were measured by the corresponding ELISA Kits (Jiangsu Meimian Industrial Co., Ltd, China), according to manufacturer’s instruction.

**Quantitative real-time PCR (RT-qPCR）analysis**

Total RNA was extracted from the samples, and the concentration and purity of RNA were determined using NanoDrop® ND-2000. Denaturing agarose gel electrophoresis was performed to assess RNA integrity. Reverse transcription was carried out using the miRNA cDNA first-strand synthesis kit (Tiangen Biotech, Catalog: KR201), and real-time PCR reactions were conducted based on the following primer sequences.

**Table 3. Primer Sequence of miRNA-21**

| **Primer name** | | **Primer Sequence (5'to3')** | **Size** |
| --- | --- | --- | --- |
| mmu-miRNA-21 | upstream | TAGCTTATCAGACTGATGTTG | 65bp |
| mmu-miRNA-21 | downstream | GGCCAACCGCGAGAAGATG |  |
| U6 internal control | upstream | CTGCGCAAGGATGACACGCAAATT | 69bp |
| U6 internal control | downstream | GGCCAACCGCGAGAAGATG |  |

**Reference**

1. Chen BY, Lin WZ, Li YL, Bi C, Du LJ, Liu Y, Zhou LJ, Liu T, Xu S, Shi CJ *et al*: **Roles of oral microbiota and oral-gut microbial transmission in hypertension**. *J Adv Res* 2023, **43**:147-161.

2. **2. Classification and Diagnosis of Diabetes: Standards of Medical Care in Diabetes-2021**. *Diabetes Care* 2021, **44**(Suppl 1):S15-s33.

3. Fihn SD, Gardin JM, Abrams J, Berra K, Blankenship JC, Dallas AP, Douglas PS, Foody JM, Gerber TC, Hinderliter AL *et al*: **2012 ACCF/AHA/ACP/AATS/PCNA/SCAI/STS Guideline for the diagnosis and management of patients with stable ischemic heart disease: a report of the American College of Cardiology Foundation/American Heart Association Task Force on Practice Guidelines, and the American College of Physicians, American Association for Thoracic Surgery, Preventive Cardiovascular Nurses Association, Society for Cardiovascular Angiography and Interventions, and Society of Thoracic Surgeons**. *J Am Coll Cardiol* 2012, **60**(24):e44-e164.

4. Chinese Medical Association Emergency Physicians Branch, Emergency and First Aid Branch of the Chinese Healthcare International Exchange Promotion Association. **Emergency Rapid Diagnosis and Treatment Guidelines for Acute Coronary Syndrome (2019)**. *Chinese Journal of Emergency Medicine* 2019; **(04)**: 421-8.

5. Collet JP, Thiele H, Barbato E, Barthélémy O, Bauersachs J, Bhatt DL, Dendale P, Dorobantu M, Edvardsen T, Folliguet T *et al*: **2020 ESC Guidelines for the management of acute coronary syndromes in patients presenting without persistent ST-segment elevation**. *Eur Heart J* 2021, **42**(14):1289-1367.

6. Lu H, Zou P, Zhang Y, Zhang Q, Chen Z, Chen F: **The sampling strategy of oral microbiome**. *iMeta* 2022, **1**(2):e23.

7. Wang JW, Kuo CH, Kuo FC, Wang YK, Hsu WH, Yu FJ, Hu HM, Hsu PI, Wang JY, Wu DC: **Fecal microbiota transplantation: Review and update**. *J Formos Med Assoc* 2019, **118 Suppl 1**:S23-s31.

8. Caspi R, Billington R, Keseler IM, Kothari A, Krummenacker M, Midford PE, Ong WK, Paley S, Subhraveti P, Karp PD: **The MetaCyc database of metabolic pathways and enzymes - a 2019 update**. *Nucleic Acids Res* 2020, **48**(D1):D445-d453.

9. Asaf R, Blum S, Roguin A, Kalet-Litman S, Kheir J, Frisch A, Miller-Lotan R, Levy AP: **Haptoglobin genotype is a determinant of survival and cardiac remodeling after myocardial infarction in diabetic mice**. *Cardiovasc Diabetol* 2009, **8**:29.

10. Jiang K, Xu Y, Wang D, Chen F, Tu Z, Qian J, Xu S, Xu Y, Hwa J, Li J *et al*: **Cardioprotective mechanism of SGLT2 inhibitor against myocardial infarction is through reduction of autosis**. *Protein Cell* 2022, **13**(5):336-359.

11. Dong J, Li Y, Xiao H, Zhang S, Wang B, Wang H, Li Y, Fan S, Cui M: **Oral microbiota affects the efficacy and prognosis of radiotherapy for colorectal cancer in mouse models**. *Cell Rep* 2021, **37**(4):109886.
